# KDM5 family of demethylases promotes CD44-mediated chemoresistance in pancreatic adenocarcinomas

**DOI:** 10.1038/s41598-023-44536-2

**Published:** 2023-10-25

**Authors:** Dan Wang, Yingjun Zhang, Zhouning Liao, Heming Ge, Cenap Güngör, Yuqiang Li

**Affiliations:** 1 Department of General Surgery, Xiangya Hospital, Central South University, Changsha, China; 2 National Clinical Research Center for Geriatric Disorders, Xiangya Hospital, Central South University, Changsha, China; 3 Hunan Engineering and Technology Research Center for Agricultural Big Data Analysis and Decision-Making, Hunan Agricultural University, Changsha, China; 4 Department of Nephrology, Xiangya Hospital, Central South University, Changsha, China; 5 Division of Translational Immunology, III, Department of Medicine, University Medical Center Hamburg-Eppendorf, Hamburg, Germany; 6 Department of General, Visceral and Thoracic Surgery, University Medical Center Hamburg-Eppendorf, Hamburg, Germany; 7 NHC Key Laboratory of Cancer Proteomics and Laboratory of Structural Biology, Xiangya Hospital, Central South University, Changsha, China

**Keywords:** Cancer, Oncology

## Abstract

A growing body of evidence suggests that the histone demethylase-lysine demethylase 5 (KDM5) family is associated with drug resistance in cancer cells. However, it is still not clear whether KDM5 family members promote chemotherapy resistance in pancreatic ductal adenocarcinomas (PDAC). Comprehensive bioinformatics analysis was performed to investigate the prognostic value, and functional mechanisms of KDM5 family members in PDAC. The effects of KDM5 family members on drug resistance in PDAC cells and the relationship with CD44, as a stem cell marker, were explored by gene knockout and overexpression strategies. Finally, our findings were validated by functional experiments such as cell viability, colony formation and invasion assays. We found that the expression of KDM5A/C was significantly higher in gemcitabine-resistant cells than in sensitive cells, consistent with the analysis of the GSCALite database. The knockdown of KDM5A/C in PDAC cells resulted in diminished drug resistance, less cell colonies and reduced invasiveness, while KDM5A/C overexpression showed the opposite effect. Of note, the expression of KDM5A/C changed accordingly with the knockdown of CD44. In addition, members of the KDM5 family function in a variety of oncogenic pathways, including PI3K/AKT and Epithelial-Mesenchymal Transition. In conclusion, KDM5 family members play an important role in drug resistance and may serve as new biomarkers or potential therapeutic targets in PDAC patients.

## Introduction

Pancreatic cancer is often referred to as the “king of cancers” because of its highly aggressive nature and desperate prognosis^1^. Pancreatic ductal adenocarcinoma (PDAC) is the most common pathological type of this disease, with a 5-year survival rate less than 10%^2^. While radical surgical resection has improved survival to some extent, the majority of patients are unable to undergo surgery because they are already in an advanced or metastatic stage at the time of diagnosis^3^. For these patients, systemic chemotherapy is the only option to help relieve symptoms and slightly prolong survival, rather than curing the patient^4^. Currently, the role of gemcitabine in PDAC chemotherapy, either as single agent or combinatorial therapy, is widely accepted. However, frustratingly, most patients who receive gemcitabine chemotherapy eventually develop resistance. In recent years, a few studies have been conducted on the mechanism of chemoresistance in PDAC, but the prognosis of patients has not significantly improved, unfortunately^5^. Therefore, it is necessary to explore new potential mechanisms of gemcitabine chemotherapy resistance to provide patients with effective molecular diagnostic markers and therapeutic targets, ultimately improving prognosis.

The histone demethylase-lysine demethylase 5 (KDM5) protein family is a group of transcriptional regulators that interfere with chromatin to remove tri- and di-methylations of lysine 4 from histone H3. KDM5-mediated histone demethylation take place at the transcriptional start site in actively transcribed genes^6^. Changes in chromatin, including DNA structure and associated histones, are key mechanisms in transcription regulation^7^. Histones are extensively modified by covalent modifications that affect chromatin compaction and transcription factor binding, and can also impact the recruitment of proteins that recognize specific histone modifications to activate or inhibit promoter activity^8^. In mammals, the KDM5 family consists of four proteins: KDM5A, KDM5B, KDM5C, and KDM5D, each with different characteristics and functions under physiological and pathological conditions^9^. Dysregulation of the KDM5 family of genes has been associated with various diseases, including cancer, highlighting the importance of understanding KDM5 protein function in vivo^10^. KDM5A, also known as JARID1A or RBP2, was initially linked to the regulation of retinoblastoma protein (pRB) targets^11^. Additionally, KDM5A overexpression is thought to promote stress-tolerance in cancer cells^12,13^. KDM5B, or JARID1B/PLU-1, has been previously identified as an oncogene in breast, lung, and prostate cancer, and is may be associated with stem cell-like properties^14–17^. Interestingly, KDM5C, or JARID1C/SMCX, plays a dual role in different tissues; it is pro-carcinogenic in breast epithelial cells and conversely tumor suppressive in kidney cells^10^. However, KDM5D, or JARID1D/SMCY, has been reported to have a tumor suppressive function in prostate cancer^18^. In summary, KDM5 family members exhibit diverse roles depending on the tumor cell and environment. Most importantly, members of the KDM5 family may be associated with chemoresistance and cancer stem-like cells (CSCs)^10^, but their role in PDAC is not well understood, and further studies are necessary to elucidate their specific role for this deadly disease.

The role of CD44, as a non-kinase receptor, has been extensively studied and has been linked to chemoresistance and CSCs in several types of cancer, including PDAC^19^. CD44 is expressed in different isoforms generated by alternative splicing^20^. However, the specific mechanisms of action of different CD44 isoforms in PDAC still remain unclear. We therefore hypothesized that CD44 subtype-induced PDAC chemoresistance is closely associated with the expression of KDM5 family members. In this study, the possible mechanistic roles of KDM5 family members in the development of PDAC were comprehensively investigated using publicly available databases and various bioinformatics analysis techniques. In particular, KDM5A/C and CD44 were found as highly expressed in drug-resistant cells and that both, knockdown and overexpression of KDM5A/C affected the cellular tolerance to chemotherapy. Furthermore, CD44 silencing reduced the expression of KDM5A/C at the transcriptional and protein level, suggesting that KDM5A/C may act as a downstream target of CD44 to influence CD44-mediated chemoresistance. This study provides new insights into the mechanisms underlying drug resistance in PDAC.

## Results

### Expression of KDM5 family members in various cancers

**Figure 1:**
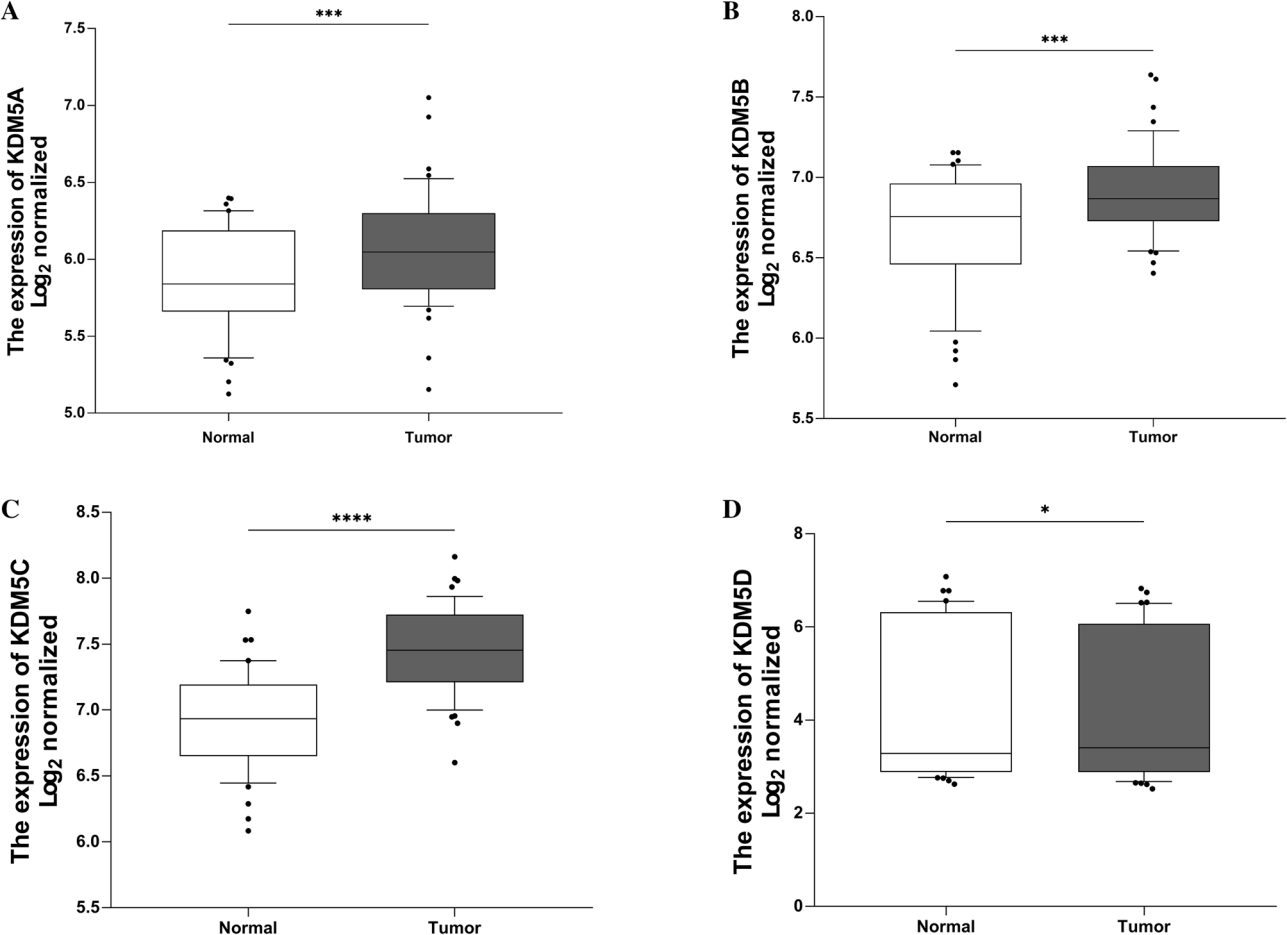
Expression of KDM5 family members in PDAC cancer tissues and paracancerous tissues. (**A**) The expression level of KDM5A; (**B**) the expression level of KDM5B; (**C**) the expression level of KDM5C; (**D**) the expression level of KDM5D. *p < 0.05; **p < 0.01; ***p < 0.001, ****p < 0.0001.

We analyzed the expression of KDM5 family members in various cancerous and normal tissues using the GEPIA website, based on data from the TCGA and GTEx databases. The results showed that transcript levels of KDM5A/B/C were significantly increased in eight different types of cancer, including PDAC, gastric adenocarcinoma (STAD) and glioblastoma multiforme (GBM). On the other hand, transcript levels of KDM5D were significantly decreased in 14 types of cancer, including PDAC, adrenocortical carcinoma (ACC) and colonic adenocarcinoma (COAD), compared to paraneoplastic or normal tissues (Supplementary Fig. 1A–D). These results suggest that KDM5 family members exhibit differential expression patterns in different types of cancer and may therefore have distinct roles in tumorigenesis. Additionally, we analyzed the expression pattern of the KDM5 family using the GEO dataset GSE28735, which compares microarray gene expression profiles of 45 matched pairs of pancreatic tumors and adjacent non-tumor tissues^21,22^. The results showed that the expression of KDM5A/B/C was significantly higher in PDAC tissues than in paracancerous tissues, while the expression of KDM5D was significantly lower in PDAC tissues (Fig. 1A–D).

### Association of KDM5 family member expression with prognosis and clinicopathological characteristics of PDAC patients

**Figure 2:**
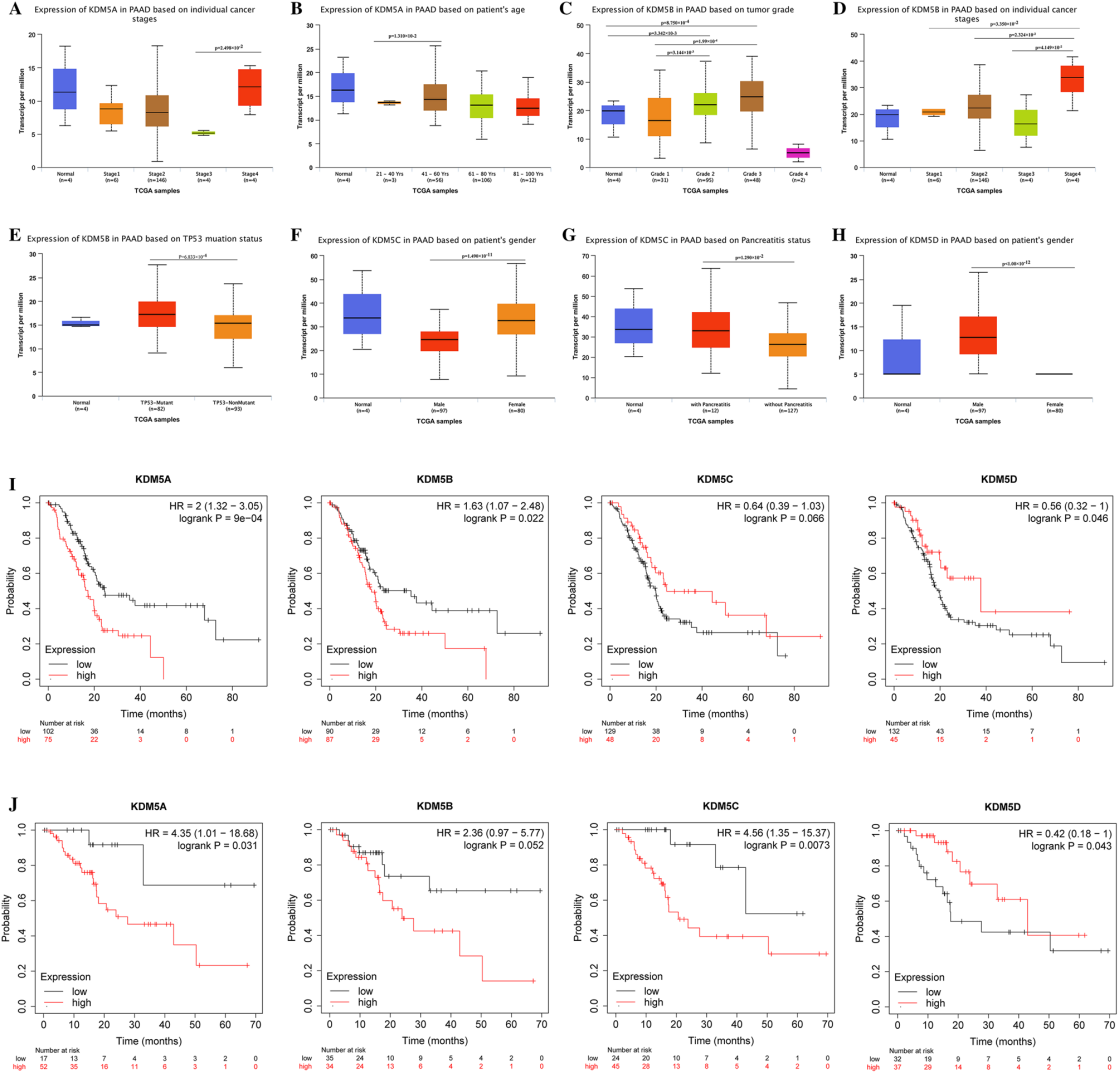
The relationship between expression levels of KDM5 family members and clinicopathologic features, and prognosis of PDAC patients. (**A**) High expression of KDM5A was significantly correlated with advanced stage; (**B**) high expression of KDM5A was significantly correlated with elderly age; (**C**) high expression of KDM5B was significantly correlated with a high tumor grade; (**D**) high expression of KDM5B was significantly correlated with an advanced tumor stage; (**E**) the expression levels of KDM5B in the *TP53* mutant PDAC tissues were significantly higher than those in the *TP53* wild type PDAC tissues; (**F**) the expression levels of KDM5C in the female PDAC tissues were significantly higher than those in the male PDAC tissues; (**G**) high expression of KDM5C was significantly correlated with a previous history of pancreatitis; (**H**) the expression levels of KDM5D in the male PDAC tissues were significantly higher than those in the female PDAC tissues; (**I**) the impact of KDM5 family expression levels on OS in PDAC patients; (**J**) the effect of KDM5 family expression levels on DFS in PDAC patients.

The expression levels of KDM5 family members in relation to the clinicopathological characteristics of PDAC patients were analyzed with data from the UALCAN database using R (version 3.6.3). These findings showed that high expression levels of KDM5A were significantly correlated with cancer stage and age (Fig. 2A, B), whereas high expression levels of KDM5B were significantly associated with tumor grade, cancer stage and *TP53* mutations (Fig. 2C–E). KDM5C was found higher expressed in female PDAC patients and a history of pancreatitis (Fig. 2F, G). However, the expression levels of KDM5D were exclusively associated with the gender of PDAC patients (Fig. 2H), and not with other clinical factors such as tumor stage, grade, and lymph node metastasis. These results suggest that KDM5 family members have distinct expression patterns and may play different roles in the development and progression of PDAC.

We also conducted a Kaplan–Meier analysis on the effect of KDM5 family member expression and on the prognosis of PDAC patients using the Kaplan–Meier Plotter database. These findings showed that elevated KDM5A/B and reduced KDM5D expression were significantly associated with poor overall survival (OS) (Fig. 2I). Moreover, the high expression of KDM5A/C and low expression of KDM5D were associated with poor disease-free survival (DFS) (Fig. 2J). In summary, this study revealed that KDM5 family members have inconsistent effects on the prognosis of PDAC patients. Overexpression of KDM5A/B/C was associated with unfavorable clinicopathological features and poor prognosis, while the opposite was shown for KDM5D.

### Functional role of the KDM5 family in PDAC

**Figure 3:**
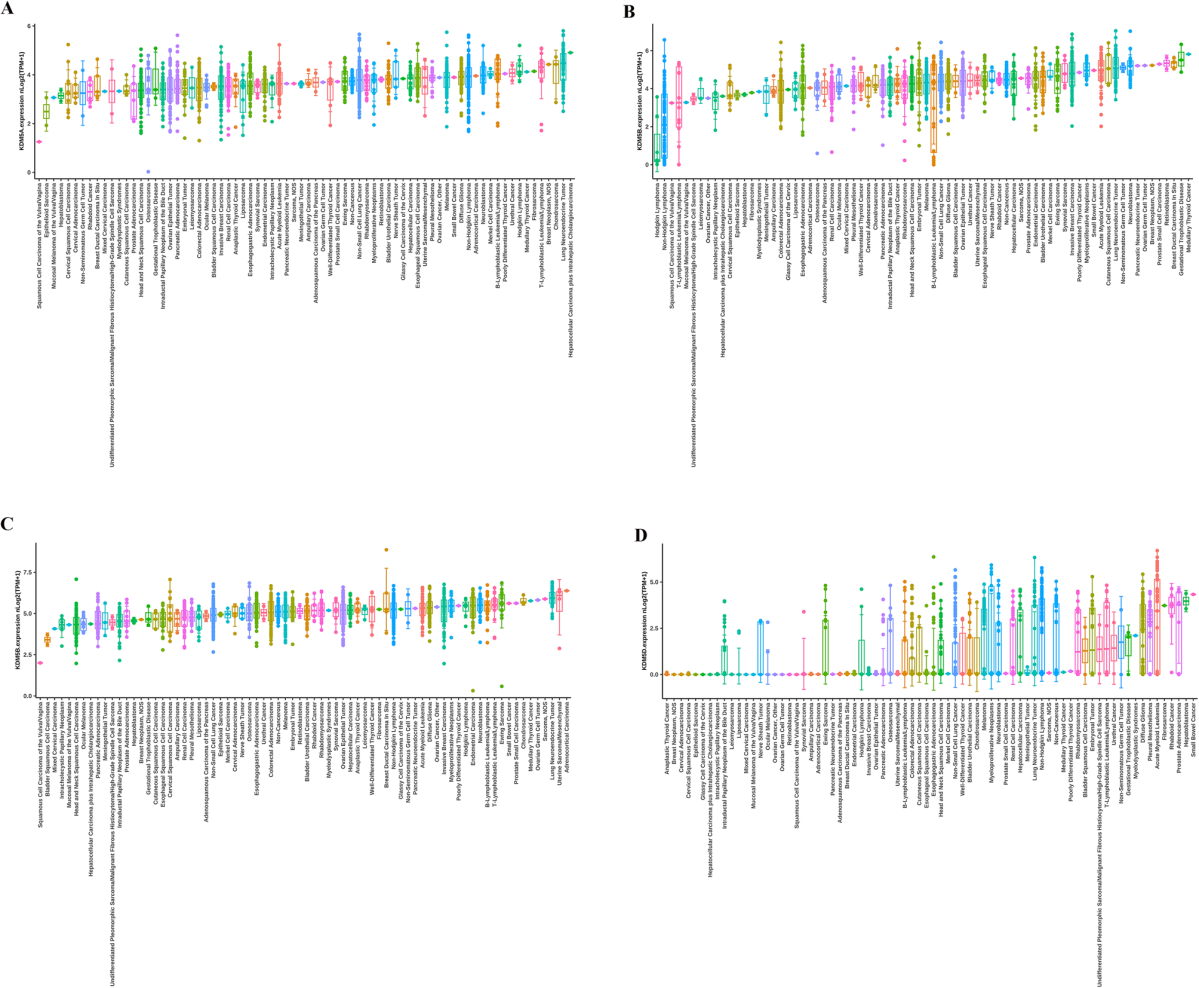
mRNA expression of KDM5 gene family in different cancer cell lines. (**A**) The expression level of KDM5A; (**B**) the expression level of KDM5B; (**C**) the expression level of KDM5C; (**D**) the expression level of KDM5D.

**Figure 4:**
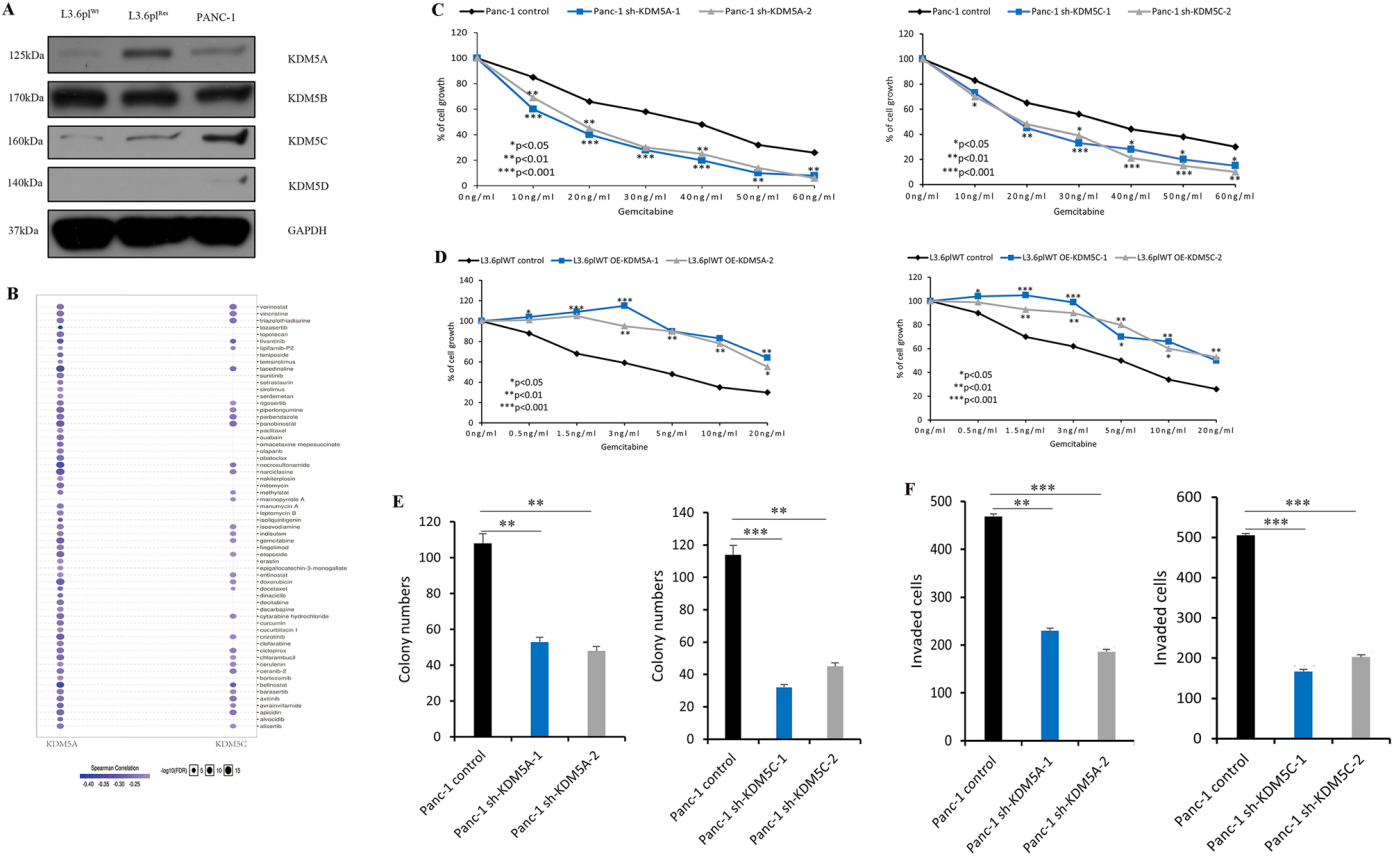
Comparison of KDM5 family protein expression and gemcitabine sensitivity in KDM5A/C stably overexpressed and knockdown PDAC cells. (**A**) Expression of KDM5 family members in different PDAC cell lines; (**B**) the expression of KDM5A/C was negatively correlated with the sensitivity of different PDAC-targeting drugs or chemotherapeutics; (**C**) cell viability assay to test drug sensitivity among two stable knockdown of KDM5A/C single clone cells (comparative analysis of the two stable knockout KDM5A/C monoclonal cell lines in their respective control groups); (**D**) cell viability assays were used to analyze drug sensitivity among two independent cell clones of KDM5A/C overexpression (comparative analysis of the two stable KDM5A/C overexpression monoclonal cell lines in their respective control groups); (**E**) comparison of colony forming ability among KDM5A/C-overexpressed and stable shKDM5A/C PDAC cells; (**F**) invasion assay comparison among KDM5A/C-overexpressed and KDM5A/C knockdown cells. All experiments were repeated three times and the results were analyzed using Unpaired t test (*p < 0.05; **p < 0.01; ***p < 0.001.).

To verify the role of the KDM5 family in pan-cancer, the mRNA expression levels of KDM5 family members in various cancer cell lines were assessed by the Cancer Cell Line Encyclopedia (Supplementary data 1–4). RNA-seq data analysis showed that cell lines from lung-associated tumors, melanoma, lymphoma, and pancreatic cancer exhibited relatively high levels of KDM5A mRNA, compared to other cell lines (Fig. 3A). Notably, cell lines from PDAC also showed relatively high levels of KDM5B/C mRNA expression, but low levels of KDM5D (Fig. 3B–D). To further confirm these findings, we examined protein levels of KDM5 family members in L3.6pl^Wt^, L3.6pl^Res^, and PANC-1 cells. As shown, protein analyses further confirmed the heterogenous expression levels of KDM5 family members in PDAC cells (Fig. 4A). Importantly, we found that the expression of KDM5A/C was significantly higher in gemcitabine-resistant cells (L3.6pl^Res^) compared to the sensitive counterpart (L3.6pl^Wt^), suggesting that KDM5A/C may be associated with PDAC drug resistance.

### The role of KDM5 family members in PDAC drug resistance

The relationship between the expression levels of KDM5 family members and therapeutic sensitivity to various drugs and targeted agents was analyzed using the GSCALite database. The results demonstrated a negative correlation between the expression level of KDM5A/C and the sensitivity of PDAC to various targeted or chemotherapeutic agents, including gemcitabine (Fig. 4B). Further experiments were conducted to investigate the role of KDM5A/C in promoting drug resistance in PDAC. Lentivirus-mediated stable KDM5A/C knockdown cells (shRNA) (PANC-1) and KDM5A/C-overexpression cell clones (L3.6pl^Wt^) were established, and two clonal cell lines for each cell knockdown and overexpression were selected to eliminate clonal variation (Supplementary Fig. 2). The resistance of these cells to gemcitabine was significantly reduced following KDM5A and KDM5C silencing using RNAi, respectively. In particular, gemcitabine inhibited the proliferation of shKDM5A/C cells in a dose-dependent manner, with the inhibitory effect of gemcitabine on cell proliferation becoming evident at concentrations of 10 ng/ml (Fig. 4C). Additionally, cell viability assays showed that overexpression of KDM5A/C led to significant gemcitabine resistance, compared to cells with low KDM5A/C expression, and significantly promoted gemcitabine resistance in a dose-dependent manner (Fig. 4D). In order to further confirm these findings, functional experiments such as colony formation and invasion assays were performed using KDM5A/C knockout and control cell lines. The colony formation assays revealed that KDM5A/C-depleted cells (monoclonal) showed significantly reduced colony number formation, compared to sh-control cells (Fig. 4E). Furthermore, the invasion assay demonstrated a significant reduction of invasive properties in shKDM5A/C knockdown cells, compared to sh-control cells (Fig. 4F). Overall, these results demonstrate that KDM5A/C plays a crucial role for chemotherapy resistance in PDAC cells.

**Figure 5:**
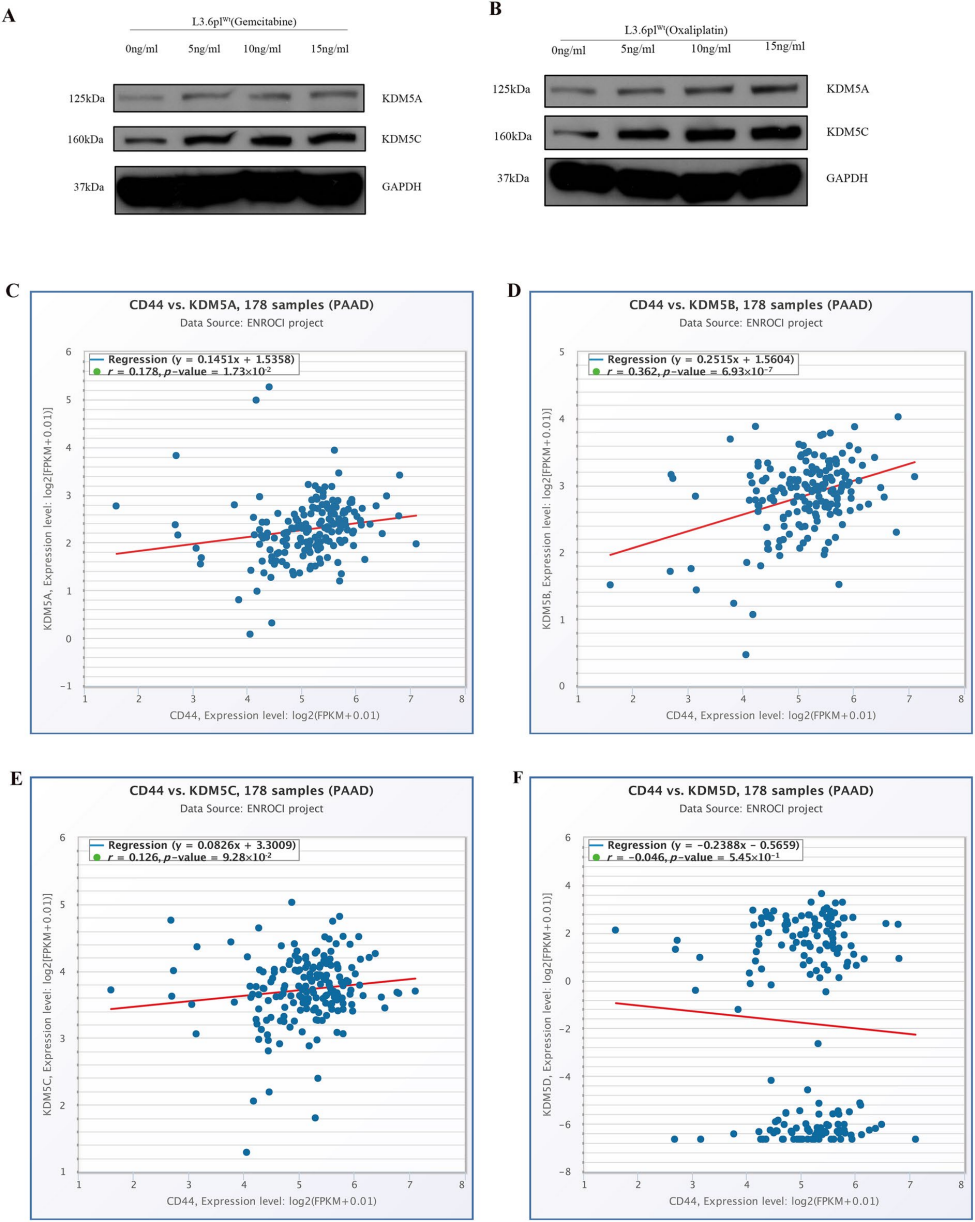
Chemotherapeutic drugs induced the expression of KDM5A/C and the co-expression pattern of KDM5 family and CD44 in PDAC. (**A**) The expression of KDM5A/C is dose-dependently induced by gemcitabine in L3.6pl^Wt^ cells; (**B**) the expression of KDM5A/C is dose-dependently induced by oxaliplatin in L3.6pl^Wt^ cells; (**C**) the correlated expression of KDM5A and CD44 from the Starbase; (**D**) the expression of KDM5B and CD44; (**E**) the expression of KDM5C and CD44; (**F**) the expression of KDM5D and CD44.

To investigate whether KDM5A/C is inducible by chemotherapy, KDM5A/C low expression cell line (L3.6pl^wt^) was treated with increasing concentrations of different chemotherapeutics (gemcitabine/Oxaliplatin) to further demonstrate an association between KDM5A/C expression and chemotherapy resistance (Fig. 5A, B) (Supplementary Fig. 3). These results manifested that KDM5A/C expression is inducible by chemotherapy in a dose-dependent manner in chemosensitive PDAC cells.

### Correlation analysis of CD44 and KDM5 family member expression in PDAC patients

**Figure 6:**
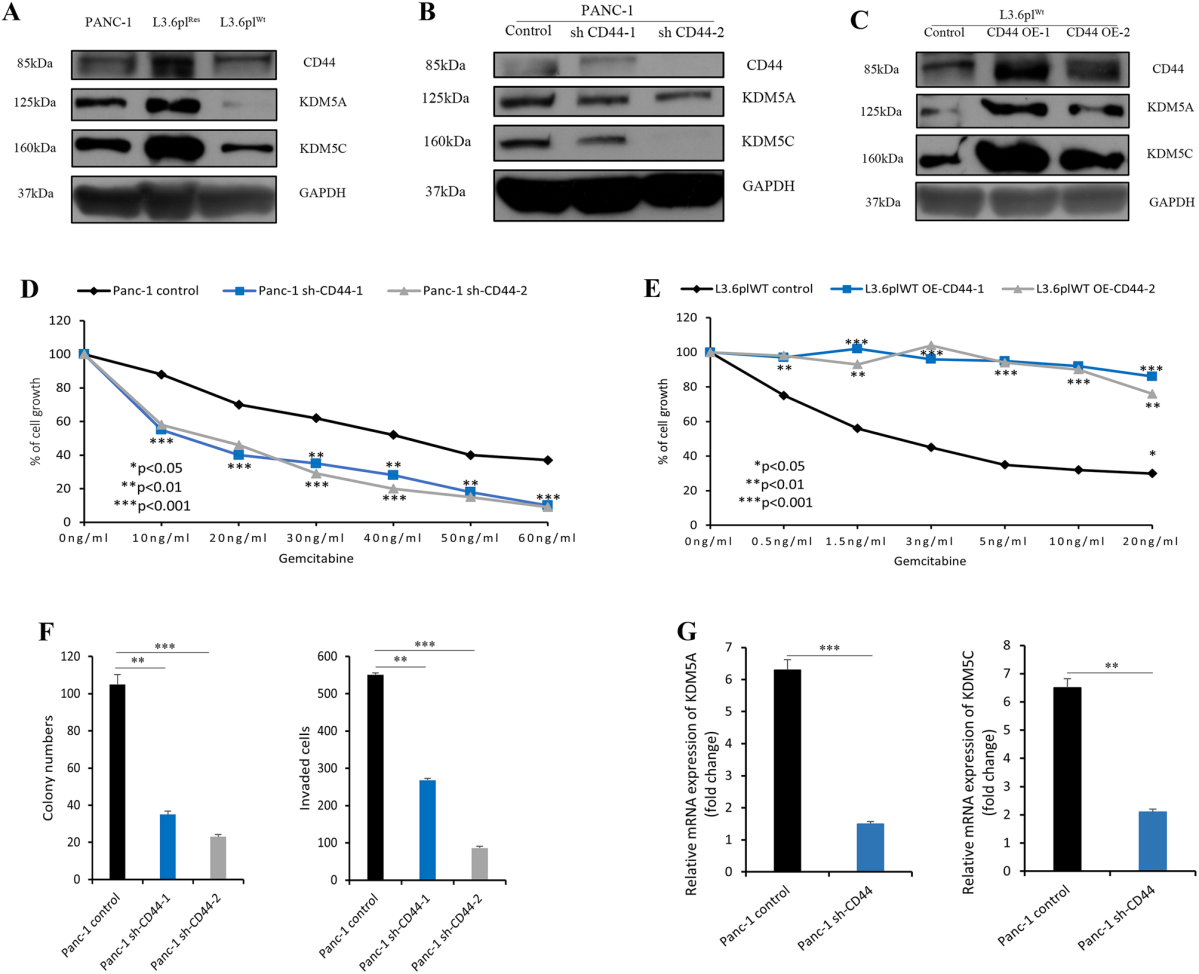
The molecular link of KDM5 family members and CD44 and its impact on chemoresistance in PDAC. (**A**) Compared with gemcitabine-sensitive PDAC cells, gemcitabine-resistant cell lines expressed higher steady-state levels of CD44 and KDM5A/C; (**B**) compared with control cells, sh-CD44 PANC-1 cells expressed lower levels of CD44 and KDM5A/C; (**C**) compared with control cells, cells with CD44 overexpression expressed higher levels of CD44 and KDM5A/C; (**D**) cell viability assay to test drug sensitivity among CD44-high cells and two stable knockdown cell clones of CD44 PANC-1 cells (Panc-1 sh-CD44-1/2 cell versus Panc-1 control cell); (**E**) cell viability assay to test drug sensitivity among CD44-low cells and two independent cell clones of CD44 overexpressed PANC-1 cells (L3.6pl^WT^ OE-CD44-1/2 cell versus L3.6pl^WT^ control cell); (**F**) colony formation (*left*) and invasion assays (*right*) showed significantly reduced colony formation and invasion in two independent cell clones of sh-CD44 PANC-1 cells. (**G**) Real-time RT-PCR results of KDM5A/C mRNA expression levels between control and sh-CD44 PANC-1 cells. All experiments were repeated three times and the results were analyzed using Unpaired t test (*p < 0.05; **p < 0.01; ***p < 0.001.).

Initially, we identified a significant co-expression pattern of KDM5 family members and the stem cell marker CD44 in various cancers by performing Spearman correlation analysis through the starBase V3.0 website (Table 1). An in-depth expression analysis using 178 PDAC patient samples revealed a significant co-expression pattern of KDM5 family members and CD44 expression in a linear regression model. In particular, KDM5A/C showed a positive correlation with CD44, whereas KDM5D showed a negative correlation with CD44 (Fig. 5C–F). These results suggested that KDM5A/C may be regulated by CD44 to influence chemoresistance in PDAC. Protein expression analyses showed elevated levels of CD44, KDM5A and KDM5C proteins in gemcitabine-resistant cells, compared to non-resistant cells. (Fig. 6A). In proof-of-principle studies, we depleted and increased CD44 expression in PANC-1 cells and L3.6pl^Wt^, respectively, and detected a corresponding decrease and increase of KDM5A/C expression in these cells, further suggesting that CD44 and KDM5A/C expressions were positively correlated in PDAC cells (Fig. 6B, C). Moreover, the results of MTT showed that PANC-1 cells that underwent CD44 knockdown were more sensitive to gemcitabine. Gemcitabine inhibited sh-CD44 cell proliferation in a dose dependent manner with 10–60 ng/ml having the most significant reduction effects on cell proliferation, compared to those with lower doses in PANC-1 sh-CD44 cells (Fig. 6D). Correspondingly, cell viability assay showed that overexpression of CD44 drove higher gemcitabine resistance than CD44 low-expressing cells. Two independent CD44 overexpression cell clones significantly promoted gemcitabine resistance in a dose dependent manner, even at low doses starting from 2.5 ng/ml to higher doses of 40 ng/ml (Fig. 6E). Colony formation ability of two independent sh-CD44 single cell clones from PANC-1 cells showed substantially decreased colony formation, compared to control cells (Fig. 6F). The invasion assays showed that silencing of CD44 is accompanied by significantly less invasive capacity, compared to control cells. (Fig. 6F). These results further indicated that CD44 and KDM5A/C expressions were positively correlated and may contributed to chemoresistance in PDAC cells. We therefore hypothesized that KDM5A/C may potentially serve as a downstream target of CD44 expression. Therefore, we initially depleted CD44 in CD44 high-expressing PANC-1 cells to test whether KDM5A/C is differentially expressed following CD44 depletion. Within our expectation, the expression of KDM5A/C mRNA was significantly reduced in sh-CD44 PANC-1 cells, compared to control cells (Fig. 6G).

**Table 1:** Spearman correlation analysis between KDM5 family members and CD44.

KDM5 family members	Cancer	Cancer full name	Sample number	Coefficient-R	*p* value
KDM5A	KIRC	Kidney renal clear cell carcinoma	535	0.255	1.52 × 10^–7^
	KIRP	Kidney renal papillary cell carcinoma	289	0.254	1.27 × 10^–5^
	COAD	Colon adenocarcinoma	471	0.249	4.35 × 10^–8^
	PRAD	Prostate adenocarcinoma	499	0.191	1.70 × 10^–5^
	LIHC	Liver hepatocellular carcinoma	374	0.181	4.44 × 10^–4^
	PDAC	Pancreatic adenocarcinoma	178	0.178	1.73 × 10^–2^
	SKCM	Skin cutaneous melanoma	471	0.164	3.43 × 10^–4^
	BLCA	Bladder urothelial carcinoma	411	0.118	1.70 × 10^–2^
	UCEC	Uterine corpus endometrial carcinoma	548	0.089	3.67 × 10^–2^
	STAD	Stomach adenocarcinoma	375	0.062	2.28 × 10^–1^
KDM5B	PDAC	Pancreatic adenocarcinoma	178	0.362	6.93 × 10^–7^
	COAD	Colon adenocarcinoma	471	0.35	5.52 × 10^–15^
	KIRC	Kidney renal clear cell carcinoma	535	0.259	1.23 × 10^–9^
	LIHC	Liver hepatocellular carcinoma	374	0.194	1.62 × 10^–4^
	KIRP	Kidney renal papillary cell carcinoma	289	0.182	1.87 × 10^–3^
	UCEC	Uterine corpus endometrial carcinoma	548	0.099	2.08 × 10^–2^
	PRAD	Prostate adenocarcinoma	499	0.062	1.66 × 10^–1^
	SKCM	Skin cutaneous melanoma	471	− 0.073	1.15 × 10^–1^
	BLCA	Bladder urothelial carcinoma	411	− 0.083	9.39 × 10^–2^
	STAD	Stomach adenocarcinoma	375	− 0.103	4.65 × 10^–2^
KDM5C	LIHC	Liver hepatocellular carcinoma	374	0.163	1.55 × 10^–3^
	KIRP	Kidney renal papillary cell carcinoma	289	0.126	3.29 × 10^–2^
	PDAC	Pancreatic adenocarcinoma	178	0.126	9.28 × 10^–2^
	PRAD	Prostate adenocarcinoma	499	0.09	4.36 × 10^–2^
	UCEC	Uterine corpus endometrial carcinoma	548	0.055	1.99 × 10^–1^
	KIRC	Kidney renal clear cell carcinoma	535	− 0.004	9.22 × 10^–1^
	BLCA	Bladder urothelial carcinoma	411	− 0.028	5.76 × 10^–1^
	STAD	Stomach adenocarcinoma	375	− 0.03	5.60 × 10^–1^
	SKCM	Skin cutaneous melanoma	471	− 0.1	2.94 × 10^–2^
	COAD	Colon adenocarcinoma	471	− 0.157	6.08 × 10^–4^
KDM5D	LIHC	Liver hepatocellular carcinoma	374	0.153	3.05 × 10^–3^
	PRAD	Prostate adenocarcinoma	499	0.133	2.84 × 10^–3^
	KIRC	Kidney renal clear cell carcinoma	535	0.128	3.06 × 10^–3^
	UCEC	Uterine corpus endometrial carcinoma	548	0.083	5.17 × 10^–2^
	COAD	Colon adenocarcinoma	471	0.026	5.72 × 10^–1^
	STAD	Stomach adenocarcinoma	375	0.023	6.60 × 10^–1^
	SKCM	Skin cutaneous melanoma	471	0.022	6.37 × 10^–1^
	KIRP	Kidney renal papillary cell carcinoma	289	0.008	8.90 × 10^–1^
	PDAC	Pancreatic adenocarcinoma	178	− 0.046	5.45 × 10^–1^
	BLCA	Bladder urothelial carcinoma	411	− 0.065	1.86 × 10^–1^

### Enrichment analysis of KDM5 family members and establishment of PPI network

**Figure 7:**
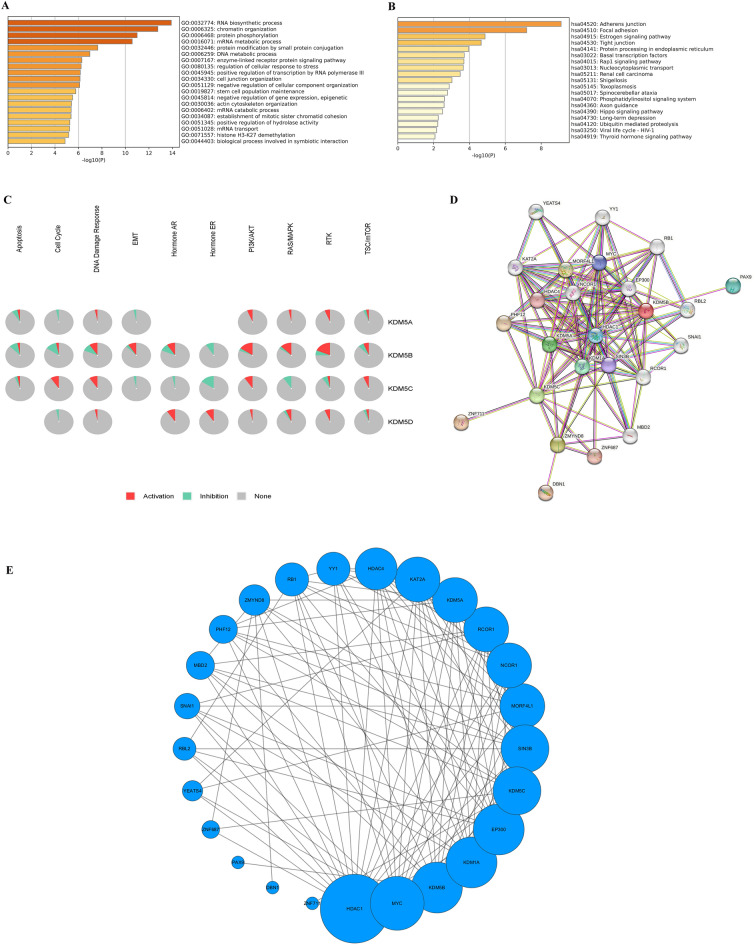
Gene enrichment analysis, and PPI network construction of KDM5 family in PDAC. (**A**) The GO enrichment of the KDM5 family and its 600 co-expressed genes involved in different biological processes (BP); (**B**) the KEGG enrichment of the KDM5 family and its 600 co-expressed genes; (**C**) KDM5 family plays an activating role in a variety of oncogenic pathways; (**D**, **E**) HDAC1 plays an important role in the identified KDM5-related PPI network.

We performed GO and KEGG enrichment analysis of KDM5 family members and their 600 co-expressed genes based on data from the databases LinkedOmics and Metascape, to further explore their mechanism of action in PDAC. These analyses showed that KDM5 family members and their co-expressed genes were mainly enriched in biological processes, such as “RNA biosynthesis”, “chromatin organization”, and “protein phosphorylation” (Fig. 7A). The molecular functions of KDM5 family members were also analyzed and were enriched for “chromatin binding”, “cadherin binding” and “transcription factor binding” (Supplementary Fig. 4). Cellular component analyses revealed that KDM5 family members were mainly enriched in “cell–cell junction”, “transcription regulator complex”, and “transcription elongation factor complex” according to GO analysis (Supplementary Fig. 4B). The KEGG analysis suggested that KDM5 family members and their co-expressed genes may play a significant role in various pathways closely related to cancer, including “Rap1 signaling pathway”, “Hippo signaling pathway”, and “Phosphatidylinositol signaling system” (Fig. 7B). Further, data analysis in the GSCA database indicated that KDM5 family members may influence the development and progression of PDAC through multiple cancer-related pathways, such as the “PI3K/AKT signaling pathway”, "EMT", "RAS/MAPK signaling pathway", "RTK signaling pathway", "apoptosis", "cell cycle", "DNA damage response signaling pathway" and "TSC/mTOR signaling pathway" (Fig. 7C). In sum, these results indicated that KDM5 family members can influence PDAC development and progression through multiple important cancer-related pathways.

The genes that had the strongest PPIs with KDM5 family members were identified using the STRING database (Fig. 7D), and the associated PPI networks were visualized using Cytoscape software (Fig. 7E). Larger circles and darker colors in the PPI network indicate a higher number of PPIs associated with the relevant gene. We determined that Histone Deacetylase 1 (HDAC1) play an important role in the PPI network closely related to KDM5 family members in PDAC. Additional experiments are necessary to validate these findings.

## Discussion

Gemcitabine is an FDA-approved nucleoside analogue used as a cornerstone in the treatment of neoadjuvant, adjuvant, and palliative cases of PDAC since 1996^23^. Despite its effectiveness, high and repeated doses of gemcitabine can lead to toxicity, and the development of chemotherapy resistance remains a significant concern^24^. Drug resistance and disease relapse pose a considerable threat to survival outcomes in PDAC patients. KDM5 family members have recently gained attention in oncology research, particularly for their roles in tumor drug resistance and stem cells^10^. Although one study has analyzed the role of the KDM5 family in pancreatic cancer^25^, there is still a substantial lack of experimental validation and no focus on its role in gemcitabine resistance. This study represents the first attempt to investigate the role of KDM5 family members in drug resistance in PDAC through a combination of bioinformatics and experimental analysis.

First, the TCGA database and GEO dataset were used to identify KDM5 family members as differentially expressed candidate genes that can be used to identify potential diagnostic and therapeutic targets in PDAC. Prognostic analysis revealed that KDM5 family members were strongly associated with relevant clinicopathological factors and prognosis. A previous study has confirmed that alterations in the KDM5 gene family may affect the prognosis of patients with non-small cell lung cancer^26^. There is increasing evidence suggesting that deregulation of KDM5 family may have important phenotypic consequences in various types of cancer, including the role of KDM5 family demethylases in the emergence of drug tolerance^27^. In this context, we found that KDM5A/C expression correlated with chemoresistance by examining the expression of KDM5 family in different PDAC cell lines. To further confirm this correlation, we conducted a loss-of-function study and found that KDM5A/C downregulation significantly increased gemcitabine sensitivity in PDAC cells, compared to cells with high KDM5A/C expression. KDM5A was already shown as overexpressed in hepatocellular carcinoma, gastric cancer and glioblastoma and is thought to be required for promoting drug resistance in cancer cells^12,13,28^. Furthermore, KDM5A binds to the gene promoter of vascular endothelial growth factor (VEGF) to increase VEGF expression, thereby promoting angiogenesis that accelerates the growth of gastric cancer cells^29^. A high allelic point mutation frequency of KDM5C was detected in pediatric acute myeloid leukemia (AML), where chemoresistance occurred^30^. The co-expression analysis in this study found KDM5 family members to be closely related to CD44, and further experiments suggested that KDM5A/C may act as a downstream target of CD44 to exert gemcitabine resistance in PDAC. Previous studies have shown that CD44-positive PDAC cells exhibit a more mesenchymal cell state, rather than epithelial and are closely linked to gemcitabine resistance^19^. The cellular-mesenchymal-epithelial transition (c-Met), a known CD44 co-receptor, affects cancer growth and metastasis by mediating its downstream signaling pathways^31^. The c-Met inhibitor reduced the number of CSCs and had a synergistic inhibitory effect with gemcitabine to suppress the growth of pancreatic tumors in a xenograft mouse model^32^. Therefore, the present study findings suggest that targeting KDM5A/C, either with antibodies or compound inhibitors, may have the potential to synergistically inhibit cellular pathways important for initiation of drug resistance mechanisms in PDAC cells.

Related pathway analysis revealed that the KDM5 family is closely associated with multiple cancer-related pathways. Our study found that KDM5B activates the EMT signaling pathway, which leads to the formation of secondary metastatic lesions by promoting cell motility/invasion and chemoresistance of tumor cells^33^. In addition, KDM5 family mainly plays a stimulatory role in the TSC/mTOR, RTK, and RAS/MAPK signaling pathways. The TSC/mTOR signaling pathway is crucial for tumor angiogenesis^34^, and the RTK and RAS/MAPK pathways can promote tumor cell growth and proliferation^35,36^. Thus, KDM5 family members can also influence PDAC development and progression through multiple oncogenic pathways.

Additionally, we found that HDAC1 plays an important role in the PPI network of KDM5 family members, and its expression product interacts with a variety of proteins. HDAC1 is an important epigenetic factor that antagonizes the acetylation state of histones and non-histones^37^. The current study found that HDAC1 is closely associated with the development and progression of pancreatic cancer. Silencing of the HDAC1 gene leads to cell cycle arrest, cell growth inhibition and induction of apoptosis in colon and breast cancers^38,39^. Overexpression of HDAC1 stimulates prostate cancer cell growth^40^, and it was demonstrated that HDAC1 inhibition downregulates the expression of cell adhesion molecule E-cadherin, thereby inhibiting the migration and proliferation of PDAC cells^41^. Notably, KDM5A has been shown to interact with the HDAC complex, thereby influencing tumor chemosensitivity^42–44^. HDAC inhibitors can also restore drug sensitivity in resistant lung adenocarcinoma PC9 cells, and removal of class I/II HDACs or KDM5A from HeLa and MCF-7 cells can enhance radiosensitivity^45,46^. Thus, further investigation on the molecular role of HDAC1 in complex with the KDM5 family is crucial for understanding resistance mechanisms in PDAC.

This study investigated and found the KDM5 protein family as frequently overexpressed in drug resistant PDAC cells. Our findings suggest that targeting KDM5 family members could be a promising strategy in the treatment course of PDAC, and that these genes may also serve as molecular markers for predicting drug sensitivity. Nevertheless, further experimental in vivo studies are needed to validate KDM5-mediated chemoresistance as a potentially new therapy target in PDAC.

## Methods and materials

### Expression analysis

This study analyzed the expression levels of KDM5 family member genes in a variety of cancer and paraneoplastic tissues from the TCGA database and the GTEx database through the GEPIA website (http://gepia.cancer-pku.cn/) (accessed February 20, 2023)^47^. Additionally, we used the GEO dataset (GSE28735) from the GEO database (https://www.ncbi.nlm.nih.gov/gds) to analyze the expression of KDM5 family members in PDAC^22^. The expression levels of KDM5 family member genes in PDAC patients with different clinicopathological characteristics were analyzed using the UALCAN database (http://ualcan.path.uab.edu) (accessed on February 20, 2023)^48^.

### Survival analysis

Correlations between the expression levels of KDM5 family members and overall survival (OS) and relapse-free survival (RFS) in PDAC patients were obtained by analysis using the Kaplan–Meier Plotter database (http://www.kmplot.com) (accessed February 30, 2023)^49^.

### Cancer cell line encyclopedia database analysis

The Encyclopedia of Cancer Cell Lines (https://portals.broadinstitute.org/ccle) is a public resource containing gene expression and sequencing information for nearly 1000 human cancer cell lines created by the Broad Institute of MIT and Harvard University^50^. This resource was used to analyze the expression of mRNAs of KDM5 family members in cell lines derived from different tumor types.

### Cell culture

L3.6pl is a secondary cell line of an orthotopic mouse xenograft model^51^. The cell lines PANC-1 and L3.6pl were obtained from the ATCC (Manassas, VA) and cultured in Dulbecco's modified Eagle's medium or RPMI 1640 (Invitrogen) supplemented with 10% fetal calf serum and 200 IU/mL Pen-Strep. Gemcitabine resistant L3.6pl cells (L3.6pl^Res^) were obtained by culturing L3.6pl cells with increasing concentrations of gemcitabine over time^52^. The resistance of L3.6pl^Res^ cells to gemcitabine was about 20 times higher than that of wild type L3.6pl cells, and the concentration of gemcitabine was added at 2 μmol/L to cultured cells.

### Drug sensitivity analysis

The association of expression levels of KDM5 family members with multiple chemotherapeutics or targeted agents for PDAC was analyzed by the GSCALite database (the Spearman test)^53^.

### Transfection of HEK293T and transduction of PDAC cells

pLenti-C-Myc-DDK-P2A-Puro Lentiviral Gene Expression Vector encoding KDM5A/C were purchased from OriGene Technologies (USA). Following transfection with lentiviral expression vector into 293 T cells, the supernatant containing lentivirals particles of 293 T cells were harvested and applied to PANC-1 cells for 48 h. Both shRNA vectors contained puromycin resistance as selection marker, so infected cells were successfully selected by puromycin for at least 2 weeks. The minimum concentration of puromycin (Sigma-Aldrich) for selection of PDAC cells was determined by Kill–Curve test. We also used the same method to establish CD44 knockout cell lines.

KDM5A/C Human Tagged ORF Clone were purchased from OriGene Technologies (USA). Transfection experiments were performed using Lipofectamine2000 (Invitrogen) according to manufacturer's procedures. The minimum concentration of neomycin (Sigma-Aldrich) for selection of PDAC cells was previously determined by Kill-Curve test. Stable KDM5A/C overexpressed cell lines (L3.6pl^Wt^) were obtained following neomycin selection in cell culture for about 2 weeks. The efficiency of all transfections was assessed by western blot.

### MTT assy

Cells were inoculated in 96-well plates (in triplicate) at a density of 5.0 × 10^3^ cells per well and treated with different concentrations of gemcitabine for 3 days. Then, cells were incubated with MTT solution (Abcam) (10 µL/well) and cultured at 37 °C for 3 h. Next, the absorbance of each well was measured at 450 nm using an automated microplate reader. The percentage of cell viability was calculated by the following formula: (absorbance of drug-treated samples/absorbance of untreated samples) × 100.

### Colony formation assay

PANC-1 and two cell clones of shKDM5A PANC-1 cells were counted and inoculated at a density of 500 cells/well. After 2 weeks, cells were fixed in methanol and stained with 0.1% crystal violet. Cell counting was performed under a microscope and photographed by Prism software. We repeated the experiment at least three times to calculate the standard deviation. We also used the same steps to perform KDM5C knockdown cells.

### Invasion assay

PANC-1 shKDM5A cells and PANC-1 control-cells were seeded at a density of 5 × 10^4^ cells in conventional fresh RPMI medium (500 µl/insert) in the upper chamber of the insert, while the bottom chamber was completed with complete RPMI medium containing 10% fetal bovine serum (700 µl/insert) for 24 h. Then the insert was washed twice with pre-warmed PBS. Next, 80% ethanol was used to fix cells for 15 min and 0.1% crystal violet was used to stain for 20 min. Finally, the inserts were well washed using fresh tap water and the inserts were dried with a cotton swab. Invasive cell counting was performed using an inverted microscope. At least 3 replicate discs per group and the entire experiment was repeated at least 3 times. We also used the same method to perform invasion assays with shKDM5C knockdown and control cells.

### Western blotting (WB) and reagents

Total protein was extracted from PDAC cells with RIPA buffer (Sigma) containing 1 × protease inhibitor cocktail (Roche). Subsequently, protein concentration was determined by bicinchoninic acid (BCA) protein assay (Abcam). An equal amount of protein samples (60 μg) was separated by 10% sodium dodecyl sulfate polyacrylamide gel electrophoresis (SDS-PAGE) and transferred to a nitrocellulose membrane [Bio-Rad, Trans-Blot Transfer Medium, Pure Nitrocellulose Membrane (0.45 µm)]. Next, the membranes were blocked with 5% skim milk for 1 h and incubated with primary and secondary antibodies, respectively. Chemiluminescent detection was performed with Super Signal West Dura Extended Duration Substrate (Pierce). The antibodies used in western blots were shown in supplementary table 1.

### Quantitative real-time reverse transcriptase PCR (qRT-PCR)

Total RNA was isolated by using TRIzol (Invitrogen). The dried pellet was cleaned with the RNeasy MiniElute Kit (Qiagen). RNA concentration was measured on a NanoDrop Spectrophotometer (Peqlab). Real-time RT-PCR was conducted to quantify gene expression. Total RNA (1 μg) was reverse transcribed by using the Transcriptor cDNA Kit (Roche). PCRs were carried out in a Mastercycler ep-realplex (Eppendorf). Data were analyzed according to the comparative CT method and normalized for cyclophilin expression in each sample.

#### Treatment with chemotherapeutic drugs

We exposed PDAC cells separately to increasing concentrations of gemcitabine or oxaliplatin (from 0.5 to 10 ng/ml) and cultured them for 1 week. Then western blot experiments were performed to verify the change of KDM5A/C protein expression.

### Co-expression analysis of CD44 and KDM5 family

We performed Spearman correlation analysis based on the starBase V3.0 website to analyze the co-expression of CD44 and KDM5 family members in various cancers^54^.

### Enrichment analysis

The 600 genes most closely associated with the co-expression of KDM5 family members were selected using the LinkedOmics database (http://www.linkedomics.org/) (accessed on 21th February 2023)^55^. Then, the biological processes (BP), molecular functions (MF), cellular components (CC) and Kyoto Encyclopedia of Genes and Genomes (KEGG) of KDM5 family members and their 600 co-expressed genes were visualized through the Metascape database (https://metascape.org) (accessed on 23th February 2023)^56^. Finally, the GSCALite database was used for pathway enrichment analyses of KDM5 family members (http://bioinfo.life.hust.edu.cn/web/GSCALite/) (accessed on 23th February 2023)^53^.

### Construction of functional protein–protein interactions (PPI) network

The strongest PPIs of KDM5 family members were obtained from the STRING database (https://string-db.org/) (accessed on 24th February 2023) to establish the related functional PPI network^57^. The role of different genes in the PPI network was scored using Cytoscape software (version 3.9.1)^58,59^.

### Statistical analyses

All data in this study were expressed as mean ± standard deviation and were analyzed using Prism 8 software (GraphPad, San Diego, CA, USA) for relevant statistics. A bilateral test was used, and a *P* value less than 0.05 was considered statistically significant.

## Conclusion

This study provides novel insights into the function of KDM5 family members in PDAC. In particular, we investigated the molecular link between KDM5 family members and chemoresistance in PDAC. Interestingly, KDM5A/C expression levels are regulated by CD44 in gemcitabine-resistant PDAC cells. Thus, members of the KDM5 family may serve as new biomarkers or potential therapeutic targets for reversing drug resistance in PDAC.

### Supplementary Information

Supplementary Information 1.

Supplementary Information 2.

Supplementary Information 3.

Supplementary Information 4.

Supplementary Information 5.

Supplementary Information 6.

Supplementary Information 7.

Supplementary Information 8.

Supplementary Information 9.

Supplementary Information 10.

## Data Availability

The original contributions presented in the study are included in the article/supplementary materials; further inquiries can be directed to the corresponding author.
