# Supplementary material for: KDM5 family of demethylases promotes CD44-mediated chemoresistance in pancreatic adenocarcinomas
Source: Sci Rep. 2023 Oct 25;13:18250. doi: 10.1038/s41598-023-44536-2 (PMC10600175; doi:10.1038/s41598-023-44536-2)

Figure 4A

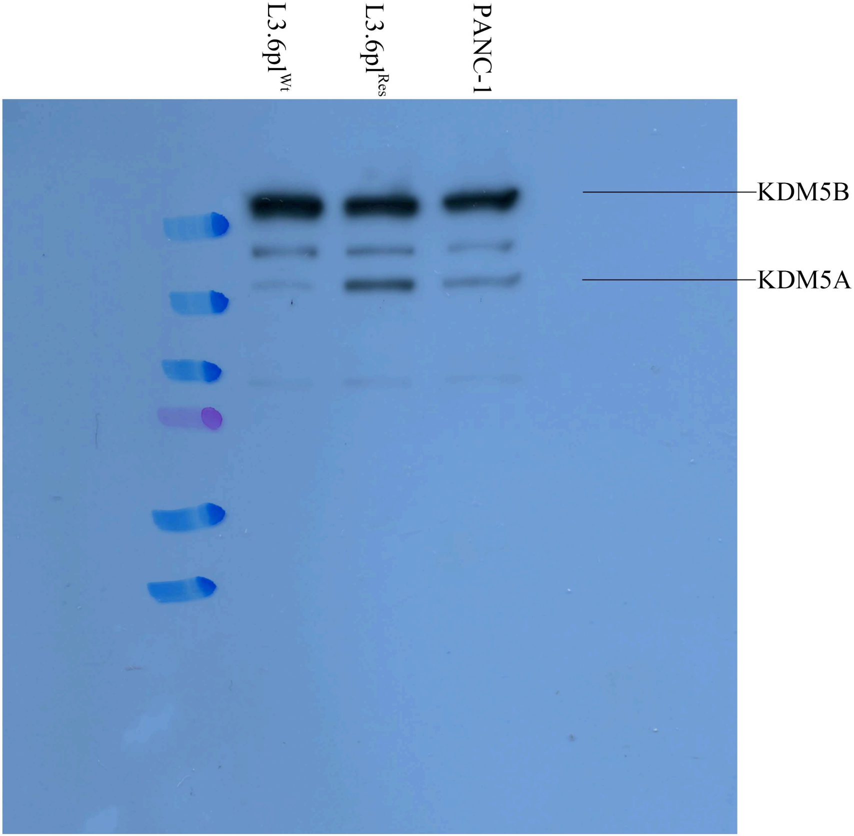

Figure 4A

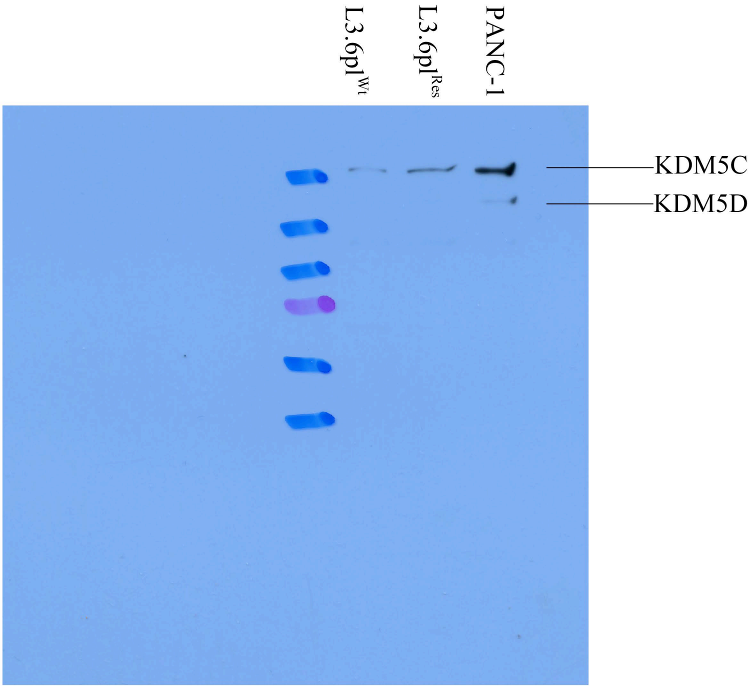

Figure 4A

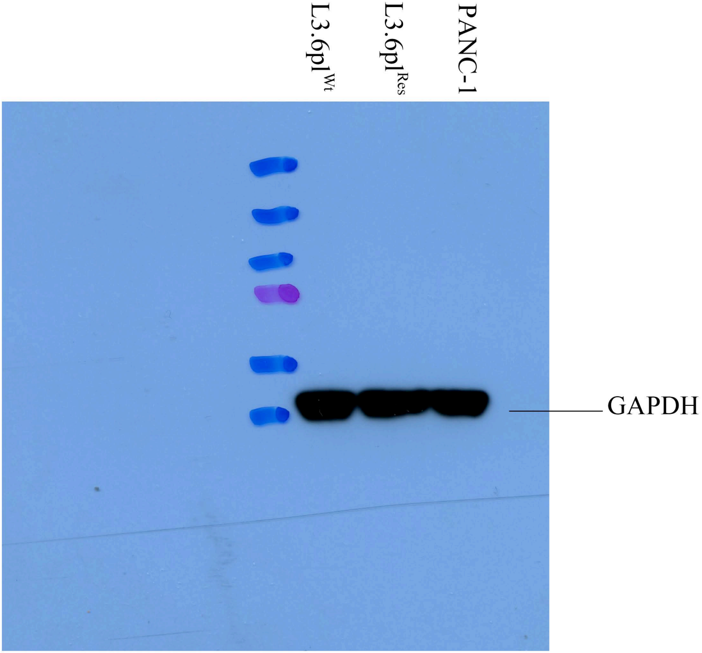

Figure 5A-B

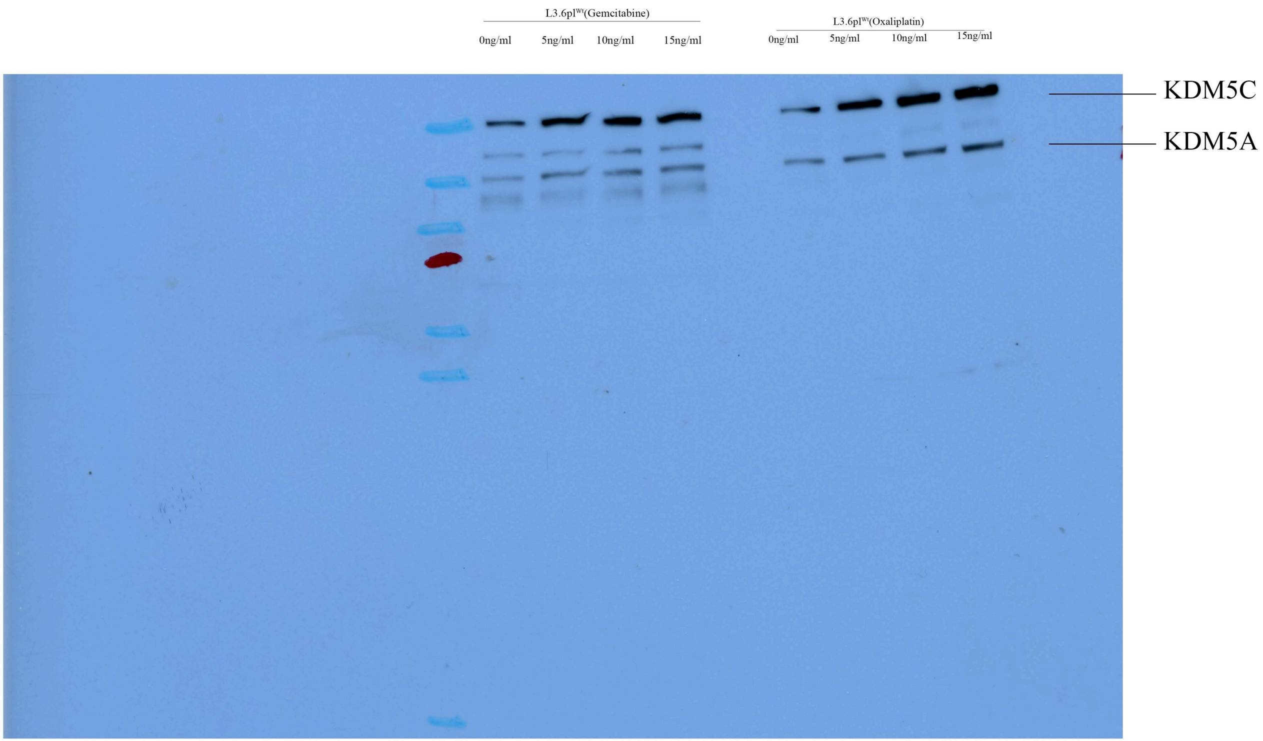

L3.6pl^{Wt}(Gemcitabine)
0ng/ml
5ng/ml
10ng/ml
15ng/ml
L3.6pl^{Wt}(Oxaliplatin)
0ng/ml
5ng/ml
10ng/ml
15ng/ml
KDM5C
KDM5A

Figure 5A-B

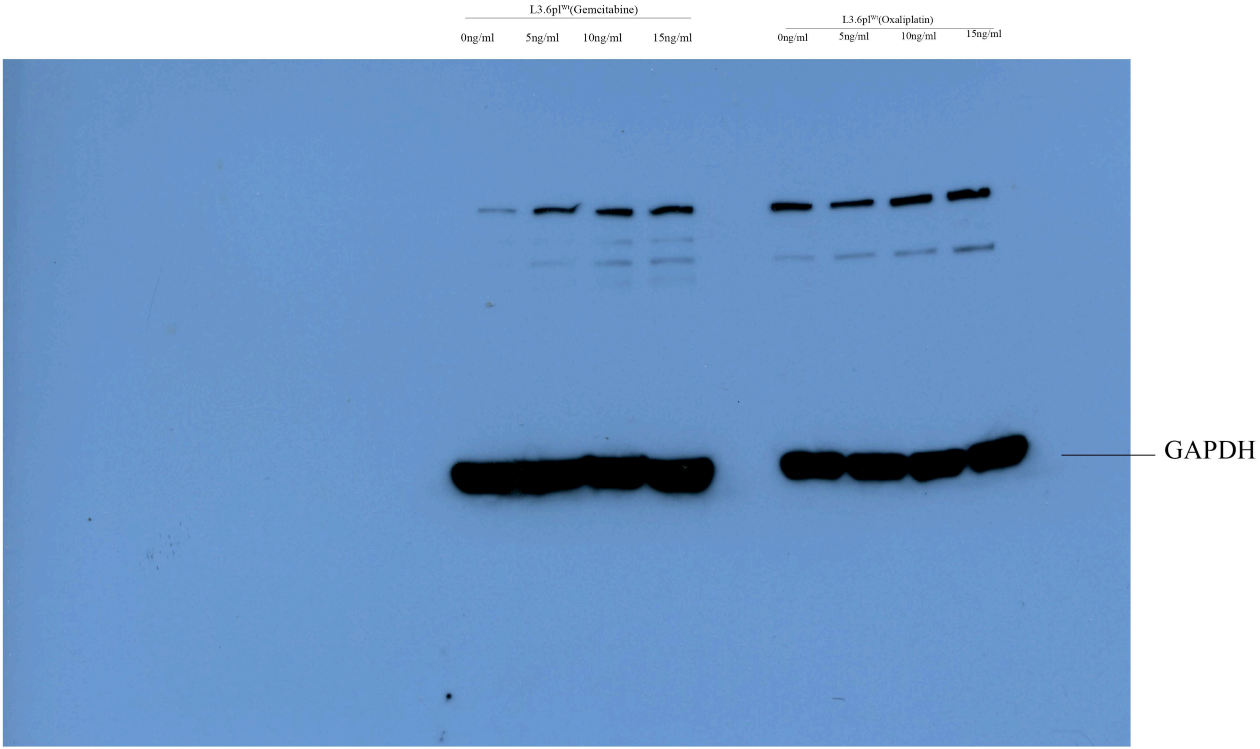
L3.6pl^wt(Gemcitabine)
0ng/ml
5ng/ml
10ng/ml
15ng/ml
L3.6pl^wt(Oxaliplatin)
0ng/ml
5ng/ml
10ng/ml
15ng/ml
GAPDH

Figure 6A-C

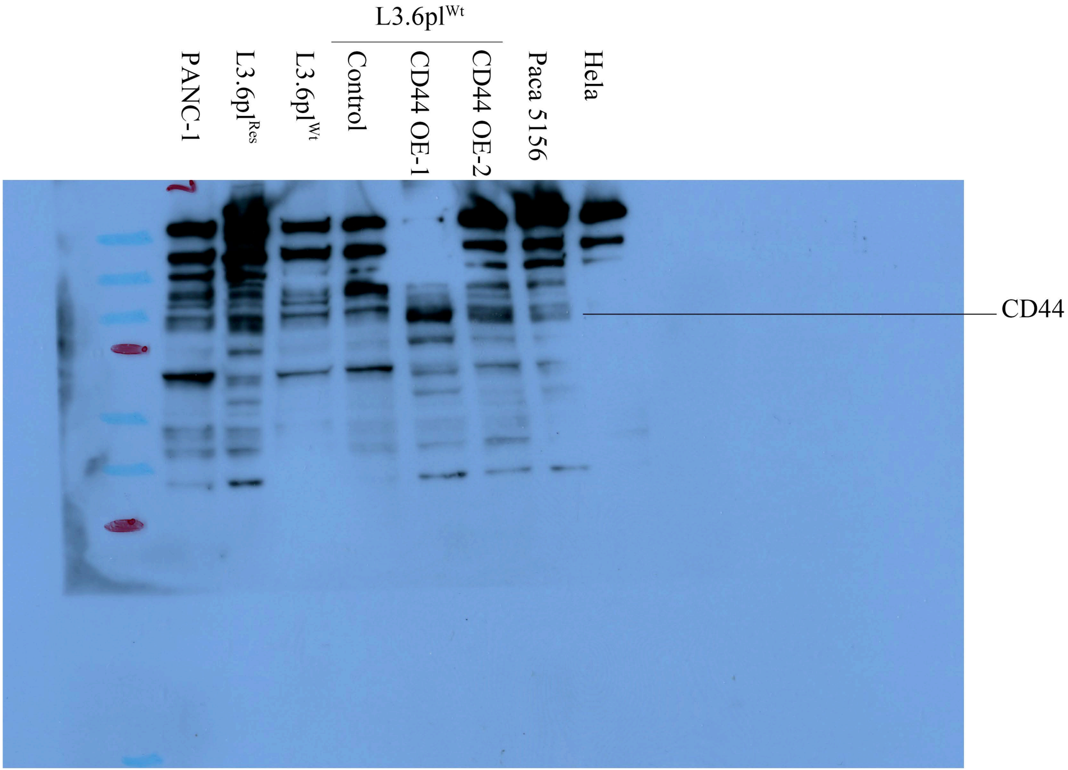

L3.6pl^{Wt}
PANC-1
L3.6pl^{Res}
L3.6pl^{Wt}
Control
CD44 OE-1
CD44 OE-2
Paca 5156
Hela
CD44

Figure 6A-C

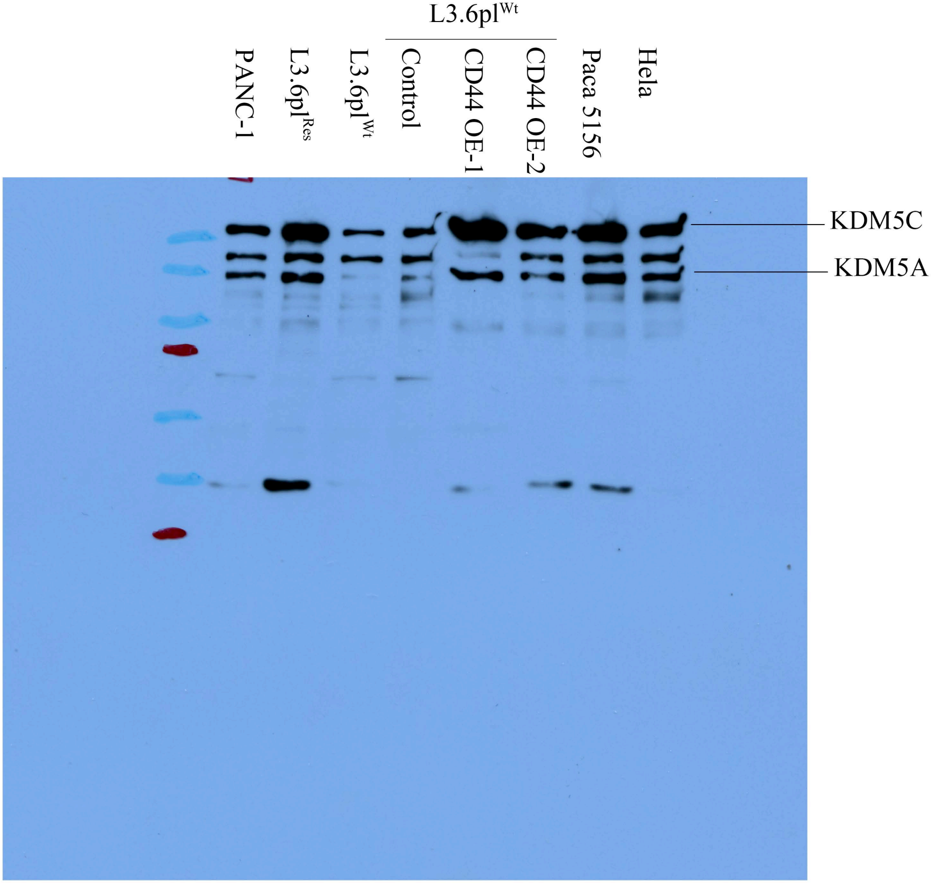
L3.6pl^Wt
PANC-1
L3.6pl^Res
L3.6pl^Wt
Control
CD44 OE-1
CD44 OE-2
Paca 5156
Hela
KDM5C
KDM5A

Figure 6A-C

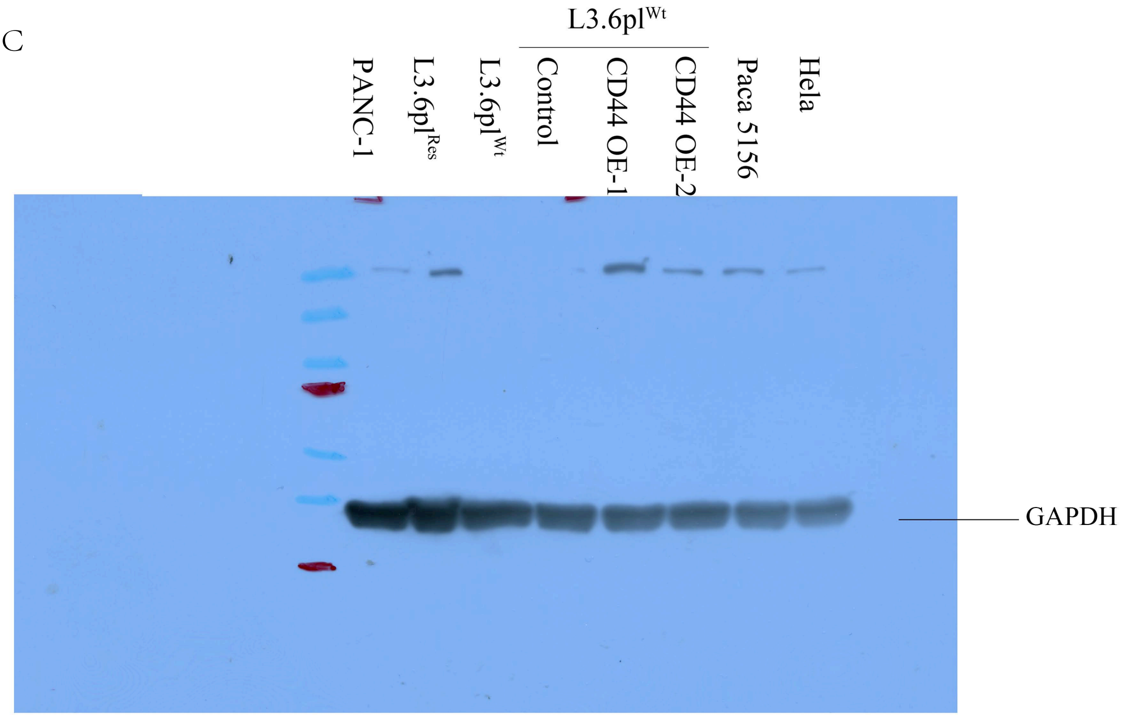
L3.6pl^Wt
PANC-1
L3.6pl^Res
L3.6pl^Wt
Control
CD44 OE-1
CD44 OE-2
Paca 5156
Hela
GAPDH

Figure 6B

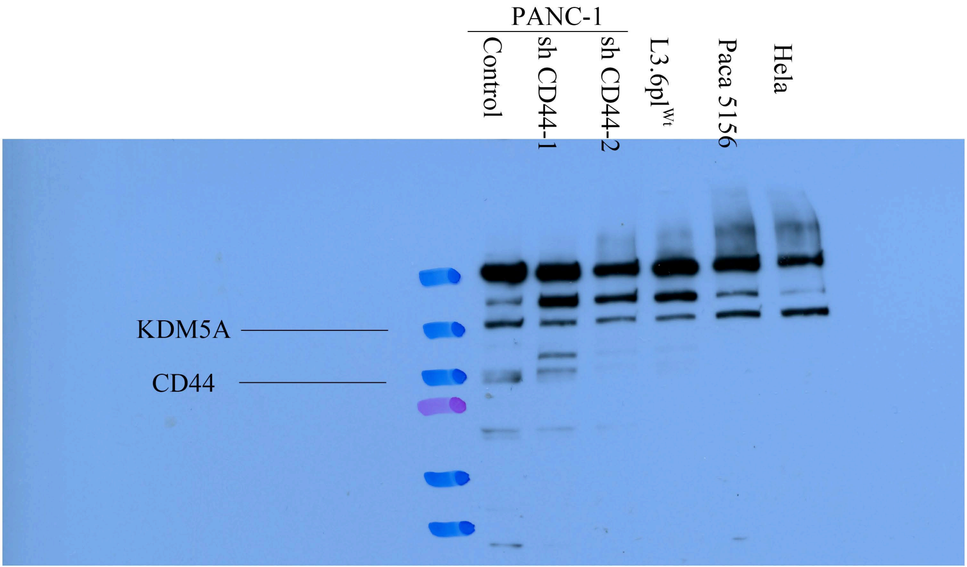
PANC-1
Control
sh CD44-1
sh CD44-2
L3.6pl^Wt
Paca 5156
Hela
KDM5A
CD44

Figure 6B

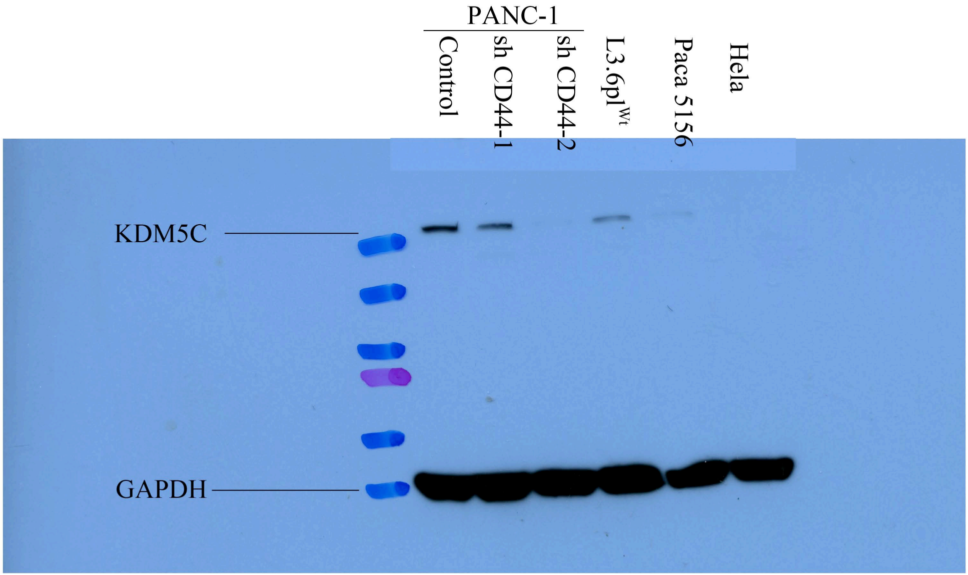
PANC-1
Control
sh CD44-1
sh CD44-2
L3.6pl^Wt
Paca 5156
Hela
KDM5C
GAPDH

Supplementary Figure 2
KDM5A OE+sh

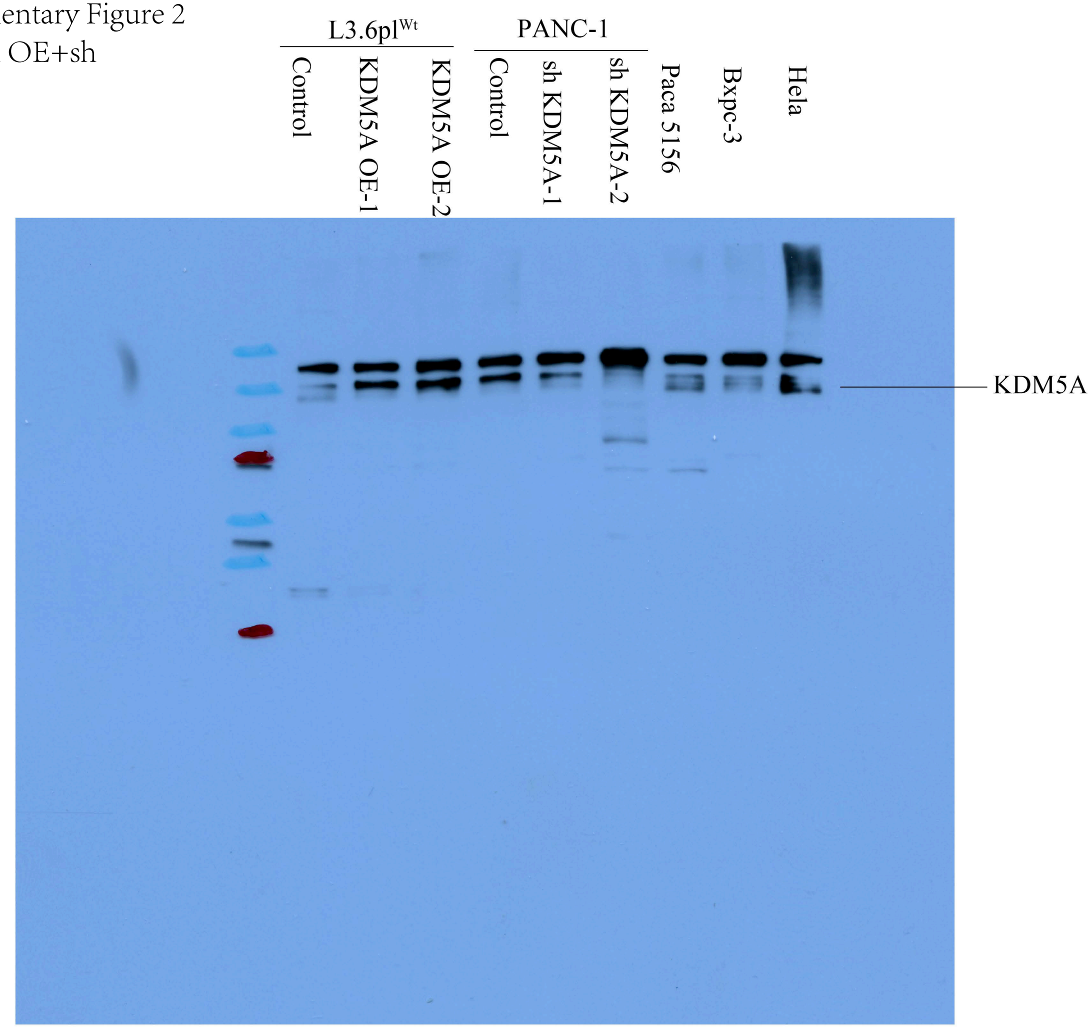

Supplementary Figure 2
KDM5A-GAPDH

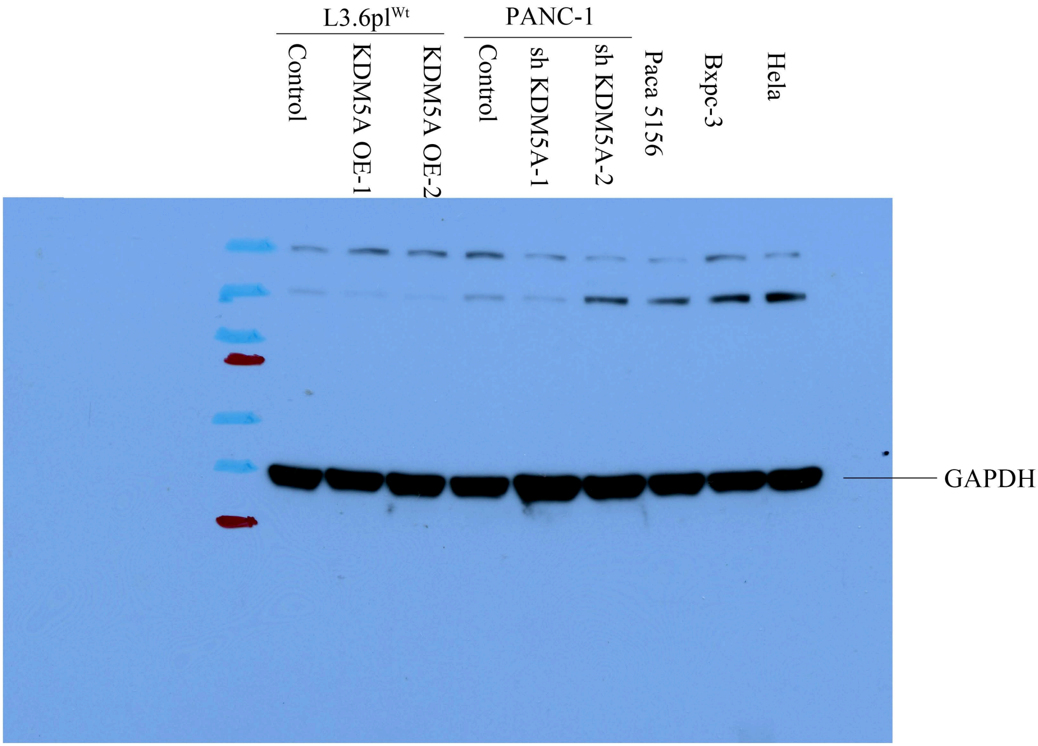

Supplementary Figure 2
KDM5C OE

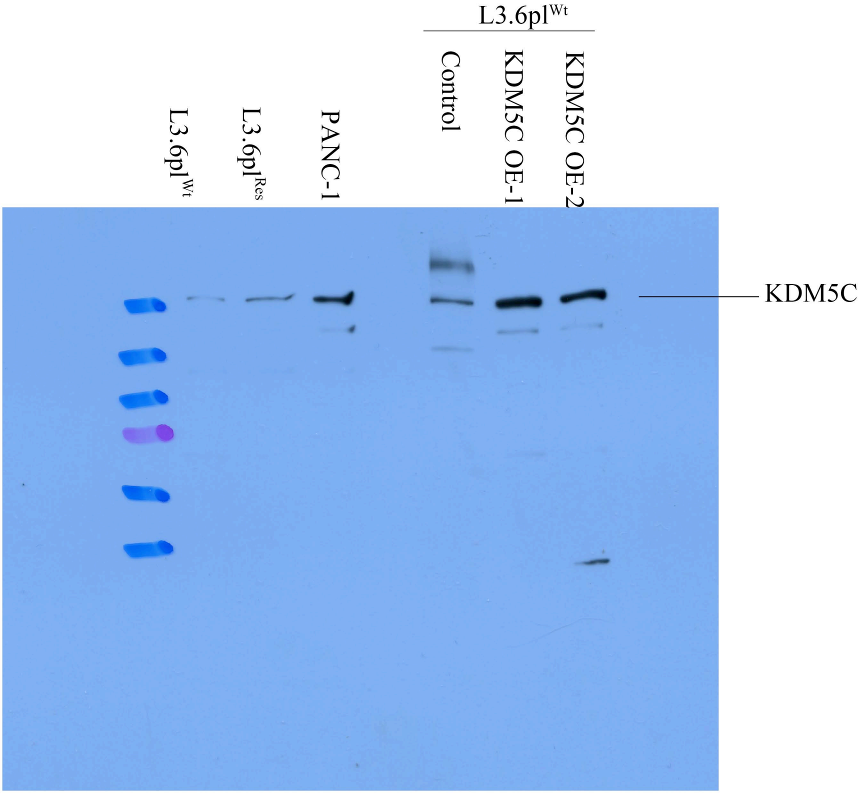

Supplementary Figure 2
KDM5C - GAPDH

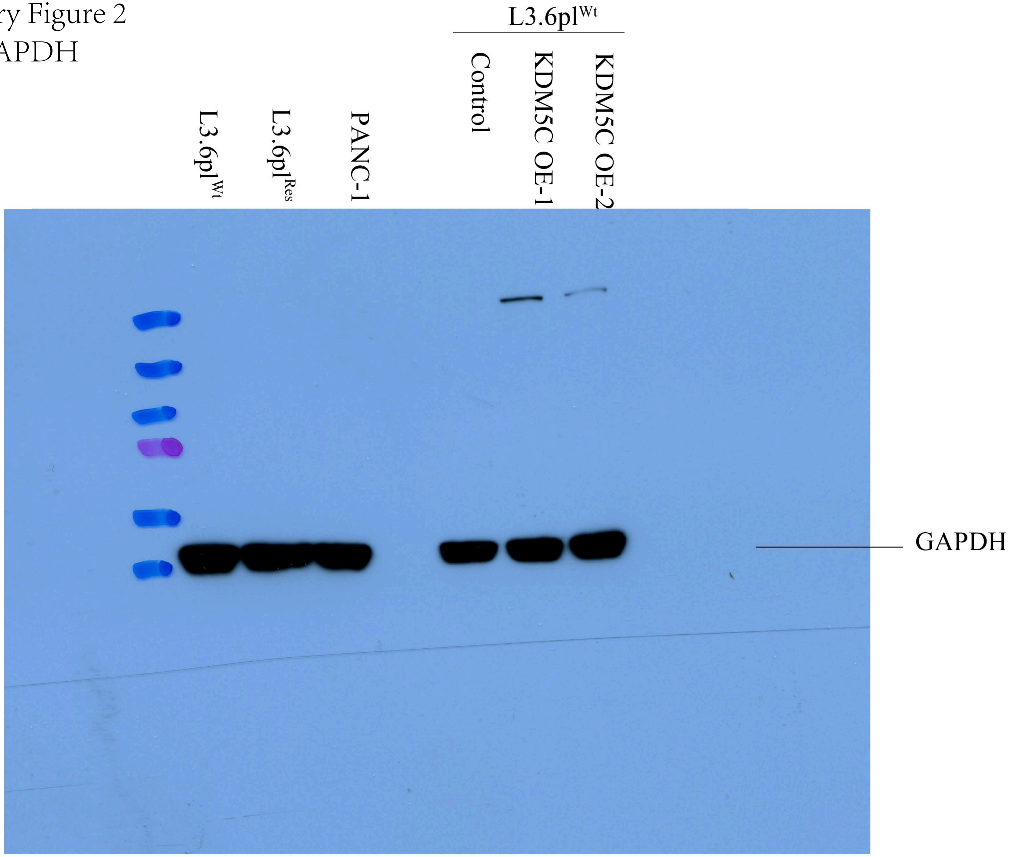

Supplementary Figure 2
sh KDM5C

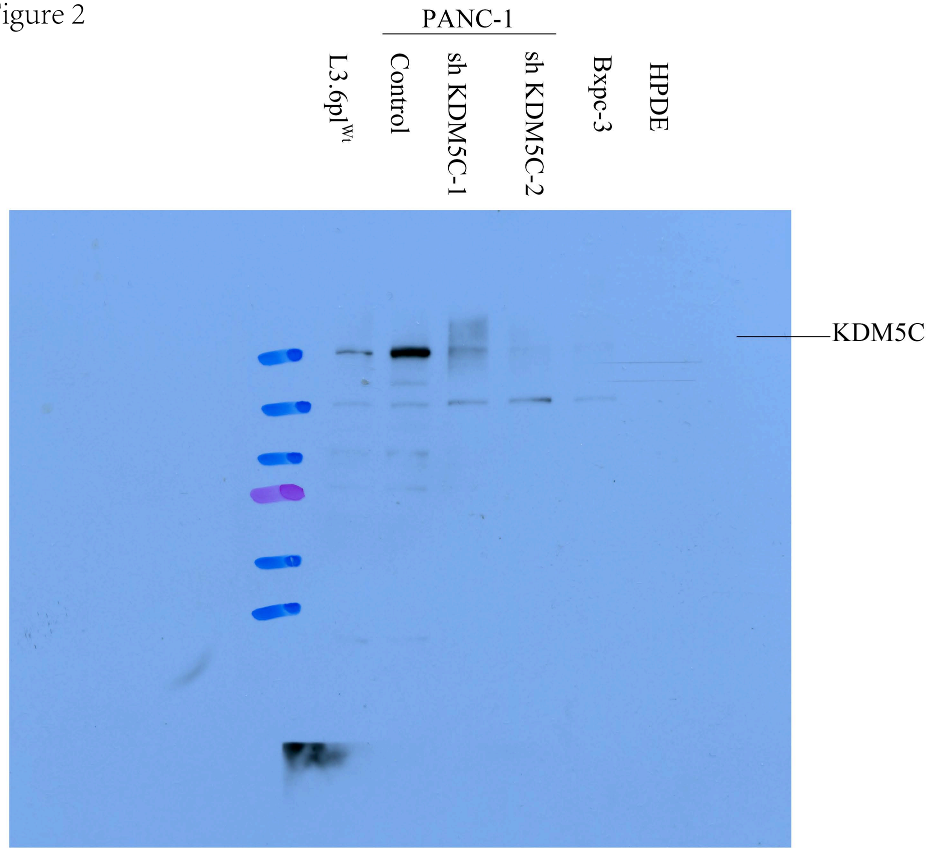

Supplementary Figure 2
sh KDM5C-GAPDH

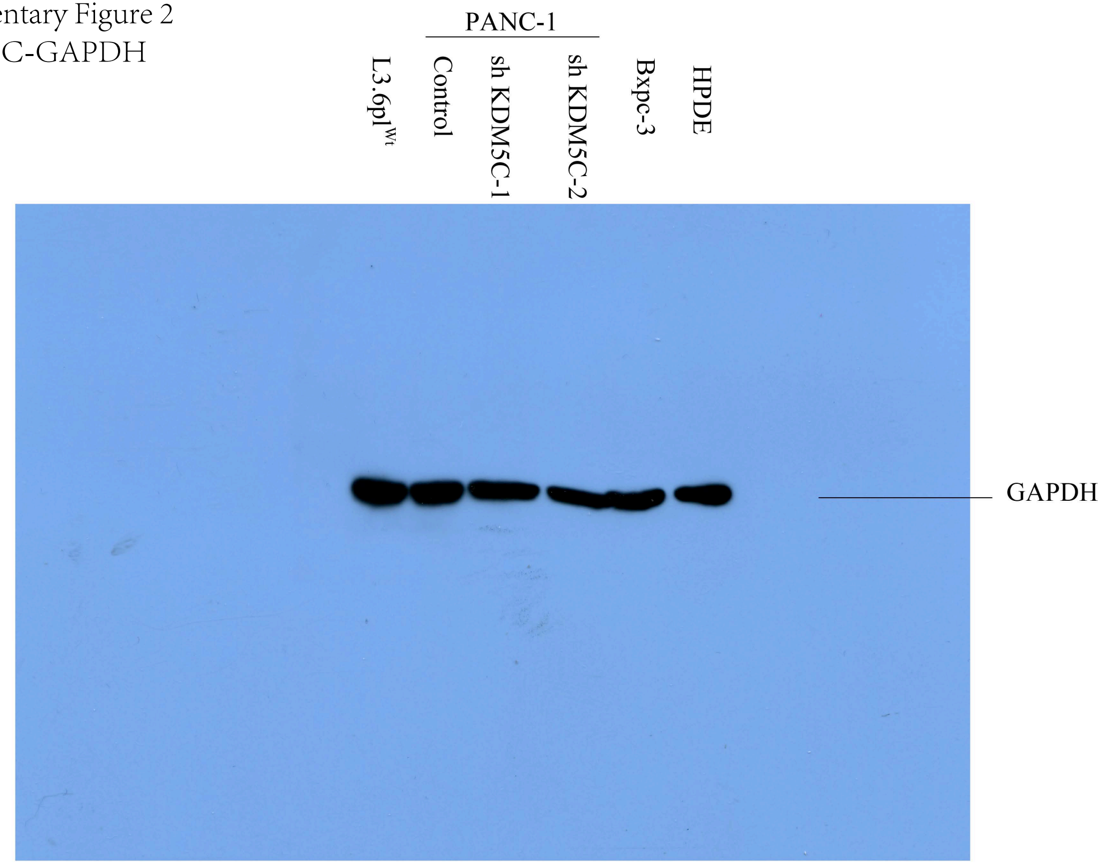

Supplement: Supplementary file 1 — Supplementary Information 1. [file 41598_2023_44536_MOESM1_ESM.pdf]
