# Supplementary figures and images for: KDM5 family of demethylases promotes CD44-mediated chemoresistance in pancreatic adenocarcinomas
Source: Sci Rep. 2023 Oct 25;13:18250. doi: 10.1038/s41598-023-44536-2 (PMC10600175; doi:10.1038/s41598-023-44536-2)

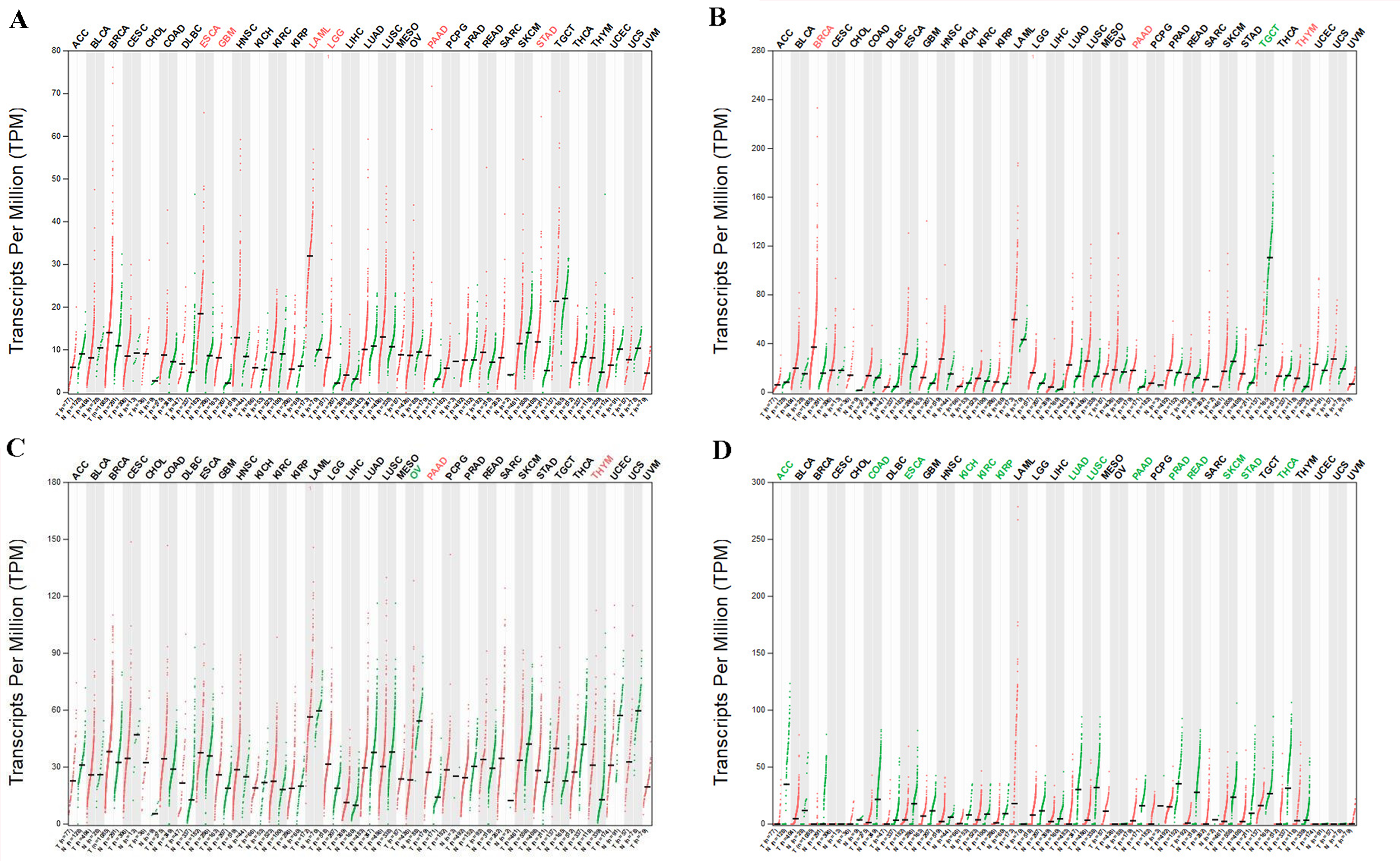

Supplement: Supplementary file 6 — Supplementary Information 6. [file 41598_2023_44536_MOESM6_ESM.tif]

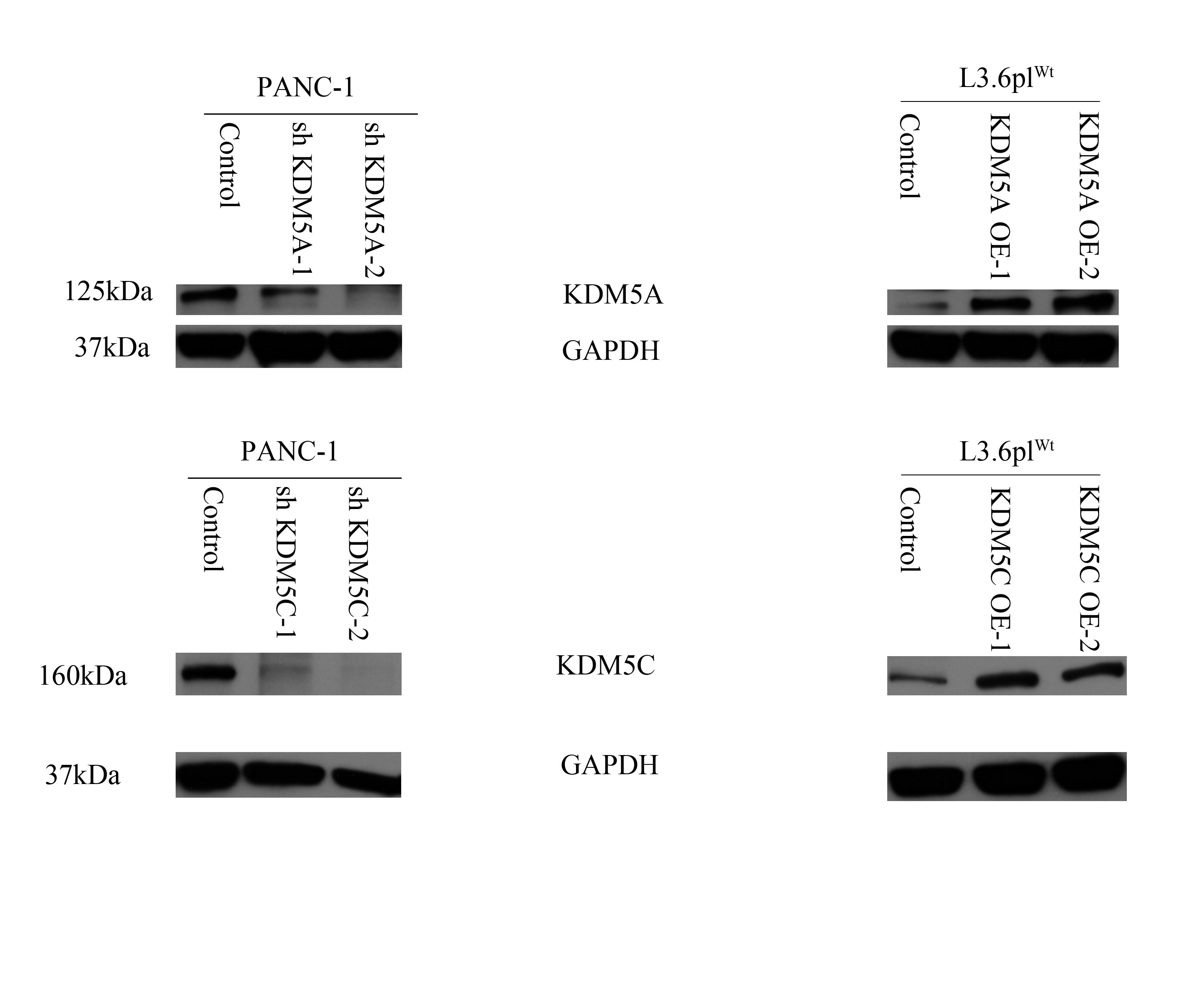

Supplement: Supplementary file 7 — Supplementary Information 7. [file 41598_2023_44536_MOESM7_ESM.tif]

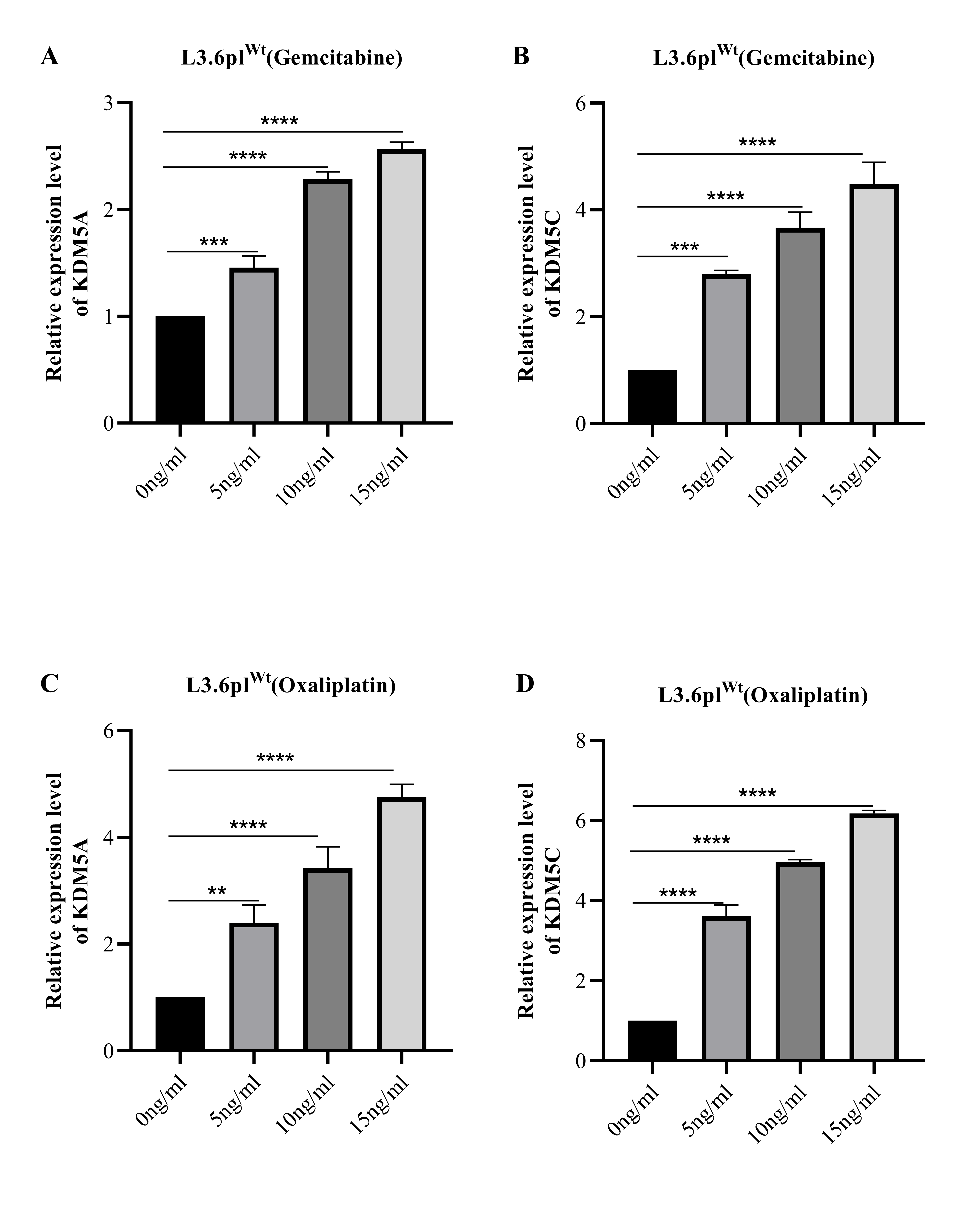

Supplement: Supplementary file 8 — Supplementary Information 8. [file 41598_2023_44536_MOESM8_ESM.tif]

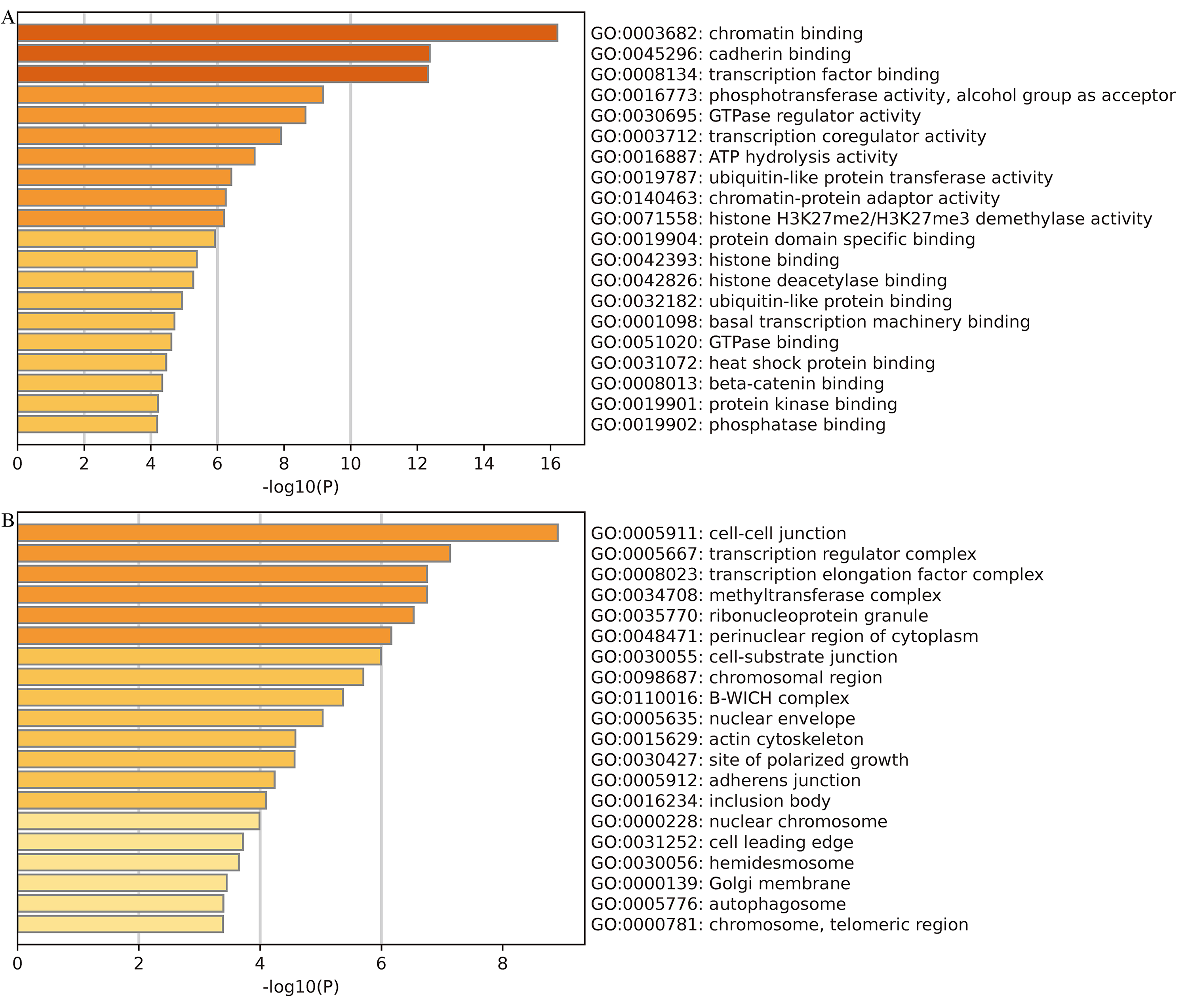

Supplement: Supplementary file 9 — Supplementary Information 9. [file 41598_2023_44536_MOESM9_ESM.tif]
