# Supplementary material for: KDM5 family of demethylases promotes CD44-mediated chemoresistance in pancreatic adenocarcinomas
Source: Sci Rep. 2023 Oct 25;13:18250. doi: 10.1038/s41598-023-44536-2 (PMC10600175; doi:10.1038/s41598-023-44536-2)
Supplement: Supplementary file 10 — Supplementary Information 10. [file 41598_2023_44536_MOESM10_ESM.docx]

**Supplementary table 1.** The primary antibodies used in this study

| Antibody | Concentration | Specificity | Company |
| --- | --- | --- | --- |
| KDM5A | 1:1000(WB); | Rabbit polyclonal | Abcam |
| KDM5B | 1:1000(WB); | Rabbit polyclonal | Abcam |
| KDM5C | 1:500(WB); | Mouse polyclonal | Abcam |
| KDM5D | 1:500(WB); | Rabbit polyclonal | Abcam |
| CD44 | 1:1000(WB) | Mouse monoclonal | Abcam |
| GAPDH | 1:1000(WB) | Rabbit polyclonal | Bioss |
